# Controlling polarization direction in epitaxial Pb(Zr_0.2_Ti_0.8_)O_3_ films through Nb (n-type) and Fe (p-type) doping

**DOI:** 10.1038/s41598-022-04802-1

**Published:** 2022-01-14

**Authors:** Cristina Florentina Chirila, Viorica Stancu, Georgia Andra Boni, Iuliana Pasuk, Lucian Trupina, Lucian Dragos Filip, Cristian Radu, Ioana Pintilie, Lucian Pintilie

**Affiliations:** National Institute of Materials Physics, Atomistilor 405A, 077125 Magurele, Ilfov Romania

**Keywords:** Materials science, Condensed-matter physics, Ferroelectrics and multiferroics

## Abstract

Fe (acceptor) and Nb (donor) doped epitaxial Pb(Zr_0.2_Ti_0.8_)O_3_ (PZT) films were grown on single crystal SrTiO_3_ substrates and their electric properties were compared to those of un-doped PZT layers deposited in similar conditions. All the films were grown from targets produced from high purity precursor oxides and the doping was in the limit of 1% atomic in both cases. The remnant polarization, the coercive field and the potential barriers at electrode interfaces are different, with lowest values for Fe doping and highest values for Nb doping, with un-doped PZT in between. The dielectric constant is larger in the doped films, while the effective density of charge carriers is of the same order of magnitude. An interesting result was obtained from piezoelectric force microscopy (PFM) investigations. It was found that the as-grown Nb-doped PZT has polarization orientated upward, while the Fe-doped PZT has polarization oriented mostly downward. This difference is explained by the change in the conduction type, thus in the sign of the carriers involved in the compensation of the depolarization field during the growth. In the Nb-doped film the majority carriers are electrons, which tend to accumulate to the growing surface, leaving positively charged ions at the interface with the bottom SrRuO_3_ electrode, thus favouring an upward orientation of polarization. For Fe-doped film the dominant carriers are holes, thus the sign of charges is opposite at the growing surface and the bottom electrode interface, favouring downward orientation of polarization. These findings open the way to obtain p-n ferroelectric homojunctions and suggest that PFM can be used to identify the type of conduction in PZT upon the dominant direction of polarization in the as-grown films.

Pb(Zr,Ti)O_3_ (PZT) ceramics and thin films are studied for to their multifunctional properties (reversible polarization under applied electric field; piezoelectricity; pyroelectricity; birefringence, etc.), making them attractive for a wide variety of applications in electronics (non-volatile memories, field effect transistors), energy (supercapacitors, photovoltaic effect), sensing (pyroelectric detectors, infrared camera), actuation (piezoelectric actuators), and others^1–3^. Properties of PZT materials, like transition temperature, dielectric constant, leakage current, can be tuned by doping or by strain enginnering^4–9^. There are plenty of studies regarding doping of PZT ceramics or thin films, in which various elements were introduced as substitutional impurities replacing either Pb or Zr/Ti ions in the crystalline lattice. Impurities were also used to control the conduction type in PZT, based on the fact that elements with valence lower than that of Pb or Ti/Zr can act as acceptors, while impurities with higher valence can act as donors^10–21^. Intrinsic defects can also exist in PZT, introducing acceptor or donor levels in the bandgap, such as Pb vacancies and oxygen vacancies, respectively^22,23^. Pb and/or O vacancies can form in response to doping to locally preserve the charge neutrality^24^. In short, doping is one way to control the PZT properties and to optimize these properties for specific applications. In the vast majority of studies, the amount of doping is above 1%, and well above this value in many cases. Some studies regarding the impact of low doping concentration on the properties of PZT were performed on ceramic-like samples, disregarding the fact that the effect of doping should be studied on samples of very good crystalline structure, preferably close to single crystal, since structural defects like grain boundaries can obscure the doping effect, leading to erroneous conclusions^20,25–28^. PZT single crystals are difficult to synthesized due to PbO volatility during growth, but epitaxial layers of very good quality can be currently grown by pulsed laser deposition (PLD) on suitable single crystal substrates like SrTiO_3_ (STO)^29,30^. Therefore, it is now possible to study the effect of dopants, acceptor and donors, on the electrical properties of epitaxial PZT layers at doping levels that are around or below 1% atomic.

Another interesting aspect reported in the literature is that the orientation of polarization in the as-grown PZT layers can be controlled by changing the substrate or the bottom electrode. For example, it was found that PZT grown on SrRuO_3_ electrode (SRO, oxide with metallic conduction) is oriented upwards (pointing towards the surface), while on (La,Sr)MnO_3_ electrode (LSMO, degenerate semiconductor with p-type conduction) is oriented downwards (pointing away from the surface)^31–33^. Similarly, polarization orientation in perovskite ferroelectrics can be controlled by changing the oxygen content or the chemistry of the surface^34–37^.

The purpose of the present study is, on one hand, to investigate the effect of 1% Fe and Nb doping on the electrical properties of epitaxial PZT films, and on the other hand, to see if the polarization orientation in the as-grown layers can be controlled by changing the doping from n-type (Nb) to p-type (Fe). The films were deposited by PLD from Fe and Nb doped targets, respectively, knowing that PLD is a method that transfers the target stoichiometry into the deposited films^29^. It was found that the electrical properties are significantly different for Fe and Nb doped PZT, respectively: the coercive field is lower in Fe doped PZT; the height of the potential barrier at the interface with SrRuO_3_ (SRO) electrodes is almost three times larger in Nb doped PZT; the concentration of charge carriers is slightly larger in Fe doped PZT, and the leakage current is with about 2 orders of magnitude larger in Fe doped PZT. These are explained by the changes introduced by the two dopants in the electronic properties of PZT. Moreover, it was found that the change of doping type affects the polarization orientation, with polarization upward in PZT-Nb and downward dominant in PZT-Fe.

## Methods

The PZT composition has a Zr/Ti ratio of 20/80, thus is in the tetragonal phase and has a large value of polarization, between 80 and 100 µC/cm^2^^30,38,39^. The Fe and Nb doped PZT layers were deposited by PLD from ceramic targets with 1% Fe and Nb doping respectively. The targets were prepared from high purity oxides of the component metal elements (details can be found in Supplemental Material-SM). A pristine PZT film was also deposited from an un-doped ceramic target, playing the role of a reference sample.

All the PZT films were deposited on single crystal STO substrates with (001) orientation hving a 20 nm thick bottom SRO electrode. The deposition was performed with a PLD workstation from Surface GmbH, having a KrF excimer laser with 248 nm wavelength and 700 mJ maximum energy. Deposition parameters are presented in SM. Top SRO/Au electrodes were deposited by PLD and magnetron sputtering, with a shadow mask, defining ferroelectric capacitors of 100 µm^2^ area.

The samples were introduced in a Lakeshore cryo-station with micro-manipulated arms and hysteresis loop (P–V), capacitance–voltage (C-V) and current–voltage characteristics (I-V) were recorded at different temperatures. The nomination of the samples is PZT-Fe and PZT-Nb for the Fe and Nb doped films, respectively, and simply PZT for the un-doped film.

An atomic force microscope (MFP 3D SA, Asylum Research) equipped with a piezo force module (PFM) was employed to obtain simultaneous high-resolution surface morphology and ferroelectric domain structure images. The images were acquired using an Olympus AC240-TM cantilevers (l = 240 μm, spring constant = 2 N/m, Pt coated) operated near resonance in the Dual AC Resonance Tracking (DART) mode at scan rates of 1 Hz.

## Results

**Figure 1:**
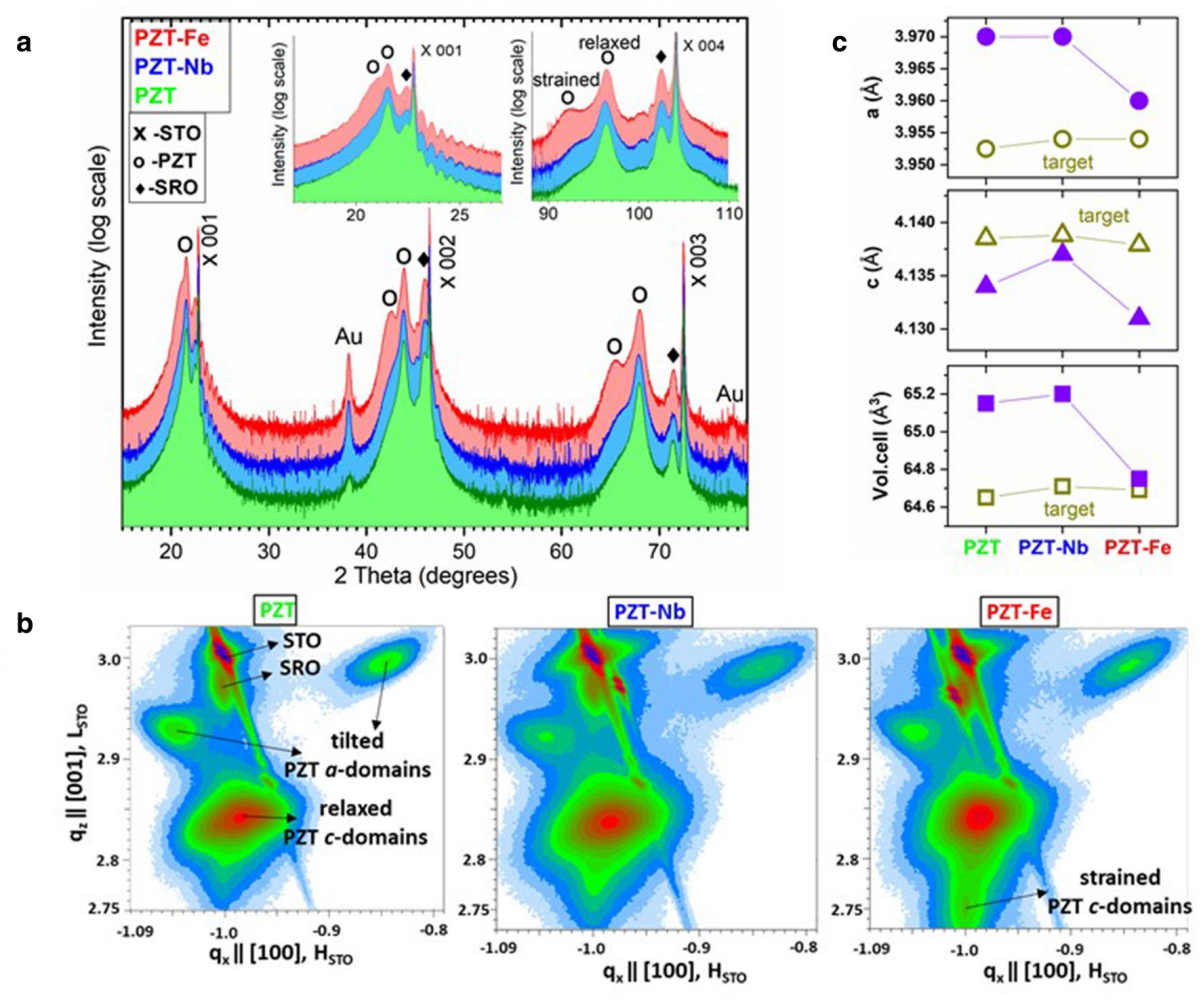
(**a**) Diffractograms (2θ-ω scans) of the PZT layer samples, with details around lines 001 and 004 in insets; (**b**) RSMs around node -103 of the pseudocubic structure; (**c**) lattice constants and volume of the unit cells obtained for the “relaxed” component of the PZT films (filled symbols) compared to those of the targets (empty symbols, see details in SM).

The results of XRD structural investigations are presented in Fig. 1a,b, and the estimated lattice constants are represented in Fig. 1c.

The structure of PZT in all samples is mainly PZT c-domains, i.e., with the *c* crystallographic axis of the tetragonal lattice perpendicular to the film surface (Fig. 1a). Inclined PZT *a*-domains are also observed in the reciprocal space maps (Fig. 1b), indicating the relaxation through *a*-domains by forming the *a/c* structure^40^, specific to PZT films above a critical thickness that may vary from sample to sample depending on the quality of the surface of the STO substrate (e.g. width and step of terraces). The fraction of *a*-domains was estimated, as detailed in SM, and it was found to be of about 3% of the whole volume of PZT in both PZT-Nb and PZT-Fe, and of about 7% in the case of un-doped PZT. Two types of *c*-domain PZT can be observed, with different values of the lattice constant *c*—perpendicular on the substrate. Most of the films have tetragonal structure with a *c* value close to the bulk one (named here “relaxed”), while the layers just above the substrate have a larger *c* value and in-plane lattice constant, *a*, close to that of the substrate (named here “strained”). The strained component has a different weight depending on the substrate structure. The fraction of the strained PZT relative to the whole *c*-domain PZT was estimated to about 3.5% in PZT-Nb and to about 5.5% in PZT-Fe, while in the un-doped PZT it is below 1%. The diffraction lines of Au are from the electrodes deposited above.

One can see from the plots in Fig. 1c that, compared to the targets, the out-of-plane parameter of the films decreases, and the in-plane one increases, which is contrary to expectations, given that the lattice mismatch with the STO substrate should induce a compressive in-plane strain. It is also noticeable that the differences between the films are larger than between the targets. The PZT-Nb film has the largest *c* lattice constant, while PZT-Fe has the smallest one, and the un-doped PZT is in between. This is true also for the targets. PZT-Fe film has also smaller in-plane lattice constant than the other films, although the *a* lattice constant of the target is the same as that of PZT-Nb. Nb doping slightly increases the volume of the unit cell, while the Fe doping reduces the volume compared to un-doped PZT. This can be explained by the fact that the Nb doping is inhibiting formation of oxygen vacancies, while Fe doping generates more oxygen vacancies compared to un-doped PZT^10,41,42^.

**Figure 2:**
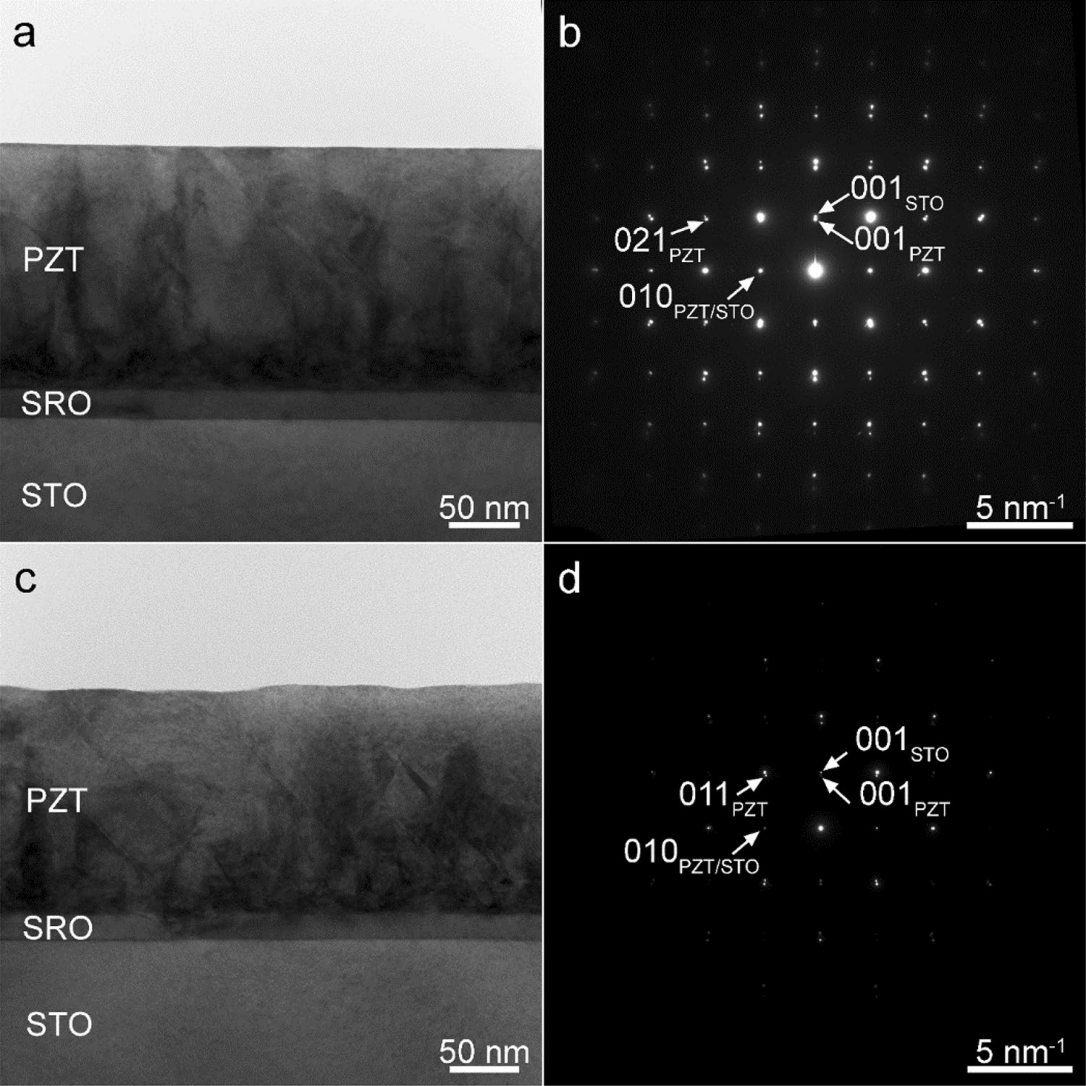
(**a**, **c**) TEM image at low magnification of the PZT:Fe/SRO/STO and PZT:Nb/SRO/STO structures and (**b**, **d**) the corresponding SAED patterns from an areas which include both the substrate and the thin film.

The TEM images for the doped-PZT/SRO/STO samples are presented in Fig. 2a,c in diffraction contrast. The TEM investigations show compact layers, with well-defined interfaces. 90^◦^ (*a/c*) domain walls are visible across the film. The PZT-Fe (Fig. 2a) layer has a thickness of, approximately, 170 nm, while the PZT-Nb (Fig. 2c) has a thickness of, approximately 162 nm. However, the PZT-Fe seems to have a smoother surface than PZT-Nb.

The thickness of the un-doped PZT layer was estimated to 192 nm from TEM images. The electron diffraction patterns from Fig. 2b,d correspond to a selected area including the PZT and SRO layers, and the STO substrate as it is suggested by the TEM images. The selected area electron diffraction (SAED) pattern in Fig. 2b,d reveal the crystallization status of the deposited layers and the orientation relationship with respect to the STO substrate. The SAED patterns reveal, for both samples, the epitaxial growth of the SRO and PZT layers on the STO(001) substrate, with (001)PZT||(101)SRO||(001)STO and (010)PZT||(010)SRO||(010)STO relations. Due to the fact that the lattice mismatch between STO and SRO is small, of about 0.5% their diffraction spots overlaps. The lattice mismatch between (010)PZT and (010)STO planes is of about 1.4% and, as a consequence, the small index spots overlap, but differences become apparent at higher Miller indices. In both cases a strain contrast can be observed in the PZT layer near the interface with the bottom SRO layer^43^.

**Figure 3:**
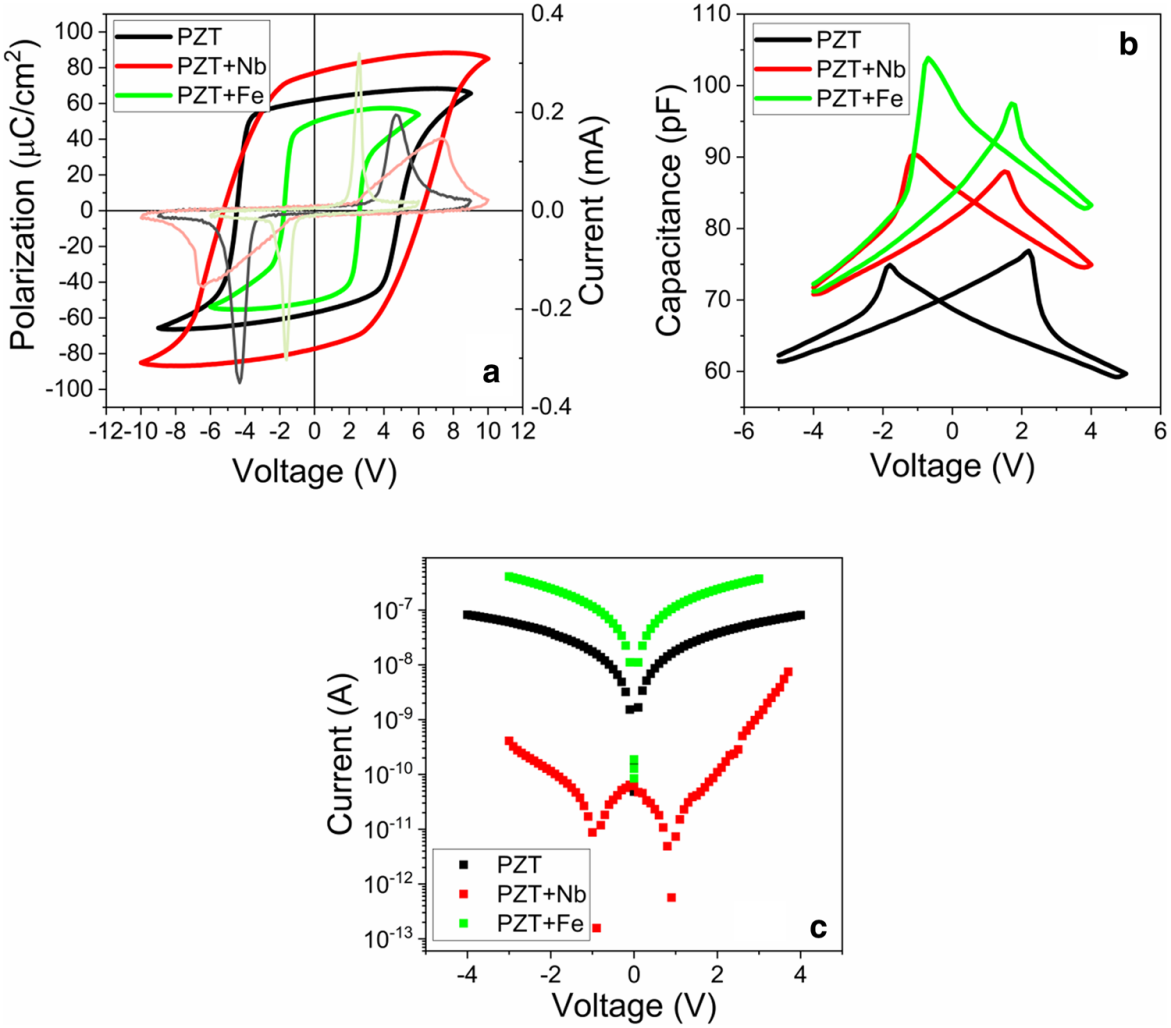
(**a**) The hysteresis P–V and I-V characteristics recorded at RT for 1 kHz frequency of high amplitude pulse; (**b**) The capacitance–voltage characteristics recorded at RT with 0.2 V a.c. signal and at 100 kHz frequency. (**c**) The current–voltage characteristics recorded for totally saturated polarization at RT.

The results of standard electrical measurements performed at room temperature are presented in Fig. 3, while the estimated values for the remnant polarization, the coercive field, the internal electric field, the dielectric constant (estimated from the capacitance measured at maximum applied voltage in C-V characteristics), the height of potential barriers at electrodes (estimated from the temperature dependent I-V measurements^44^, see details in SM), and the effective density of charge carriers (estimated from the C-V characteristics^45^, see details in SM) are given in Table 1. One has to mention that all the measurements were performed after setting the same polarization state in the samples by applying, for 10 s, a positive voltage on the top electrode, with an amplitude of 6 V for PZT-Fe, 8 V for un-doped PZT and 9 V for PZT-Nb.

**Table 1:** Values of remnant polarization (P_r_), coercive field (E_C_), internal electric field (E_int_), static dielectric constant (ε_s_), potential barrier (Φ_0_), and effective density of charge carriers (N_eff_) for the un-doped PZT, PZT-Fe and PZT-Nb films.

Sample	P_r_ (μC/cm^2^)	E_C_ (kV/cm)	E_int_ (kV/cm)	ε_S_	Φ_0_ (eV)	N_eff_ (10^18^ cm^−3^)
PZT	67	247	11.8	175	0.15 ± 0.0025	(5–7)
PZT-Fe	51	130	23.5	225	0.11 ± 0.009	(6–8)
PZT-Nb	76	338	26.5	207	0.32 ± 0.02	(3–5)

The errors in estimating the values of P_r_, E_C_, E_int_ and ε_s_ are are between 1 and 3%, the errors for potential barriers are given in the table (see details in SM).

## Discussion

**Figure 4:**
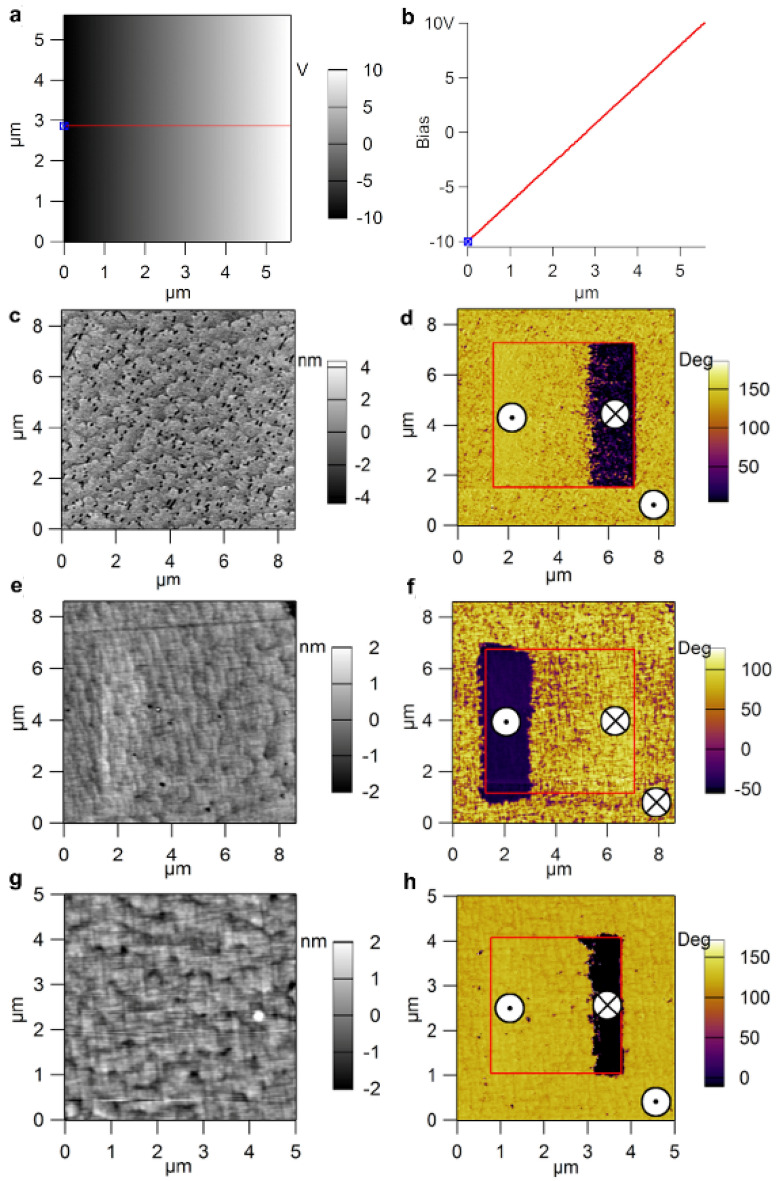
(**a**) Poling map applied for polarization switching; (**b**) section graph in the poling map image shows the applied voltage between − 10 V to + 10. AFM-PFM measurements on: (**c**, **d**) PZT-Nb; (**e**, **f**) PZT-Fe; (**g**, **h**) un-doped PZT thin films. Surface topography images are presented in (**c**, **e**, **g**), while piezoresponse phase images are shown in (**d**, **f**, **h**) after domain writing according to the poling map. The symbols are for the polarization pointing upward (black dot inside a white circle) and polarization pointing downward (cross lines in a white circle).

Analyzing the results presented in Table 1 one can see that:

- Polarization values show that un-doped PZT is in-between PZT-Fe (lowest polarization) and PZT-Nb (highest polarization), a finding that can be explained by the fact that the large leakage current in PZT-Fe, although may lead to a faster compensation of the depolarization field during polarization switching (as suggested by the very narrow current peaks in the current–voltage hysteresis loop), can also be detrimental for switching because the free carriers may screen the applied voltage during switching. This result correlates well with the fact that the PZT-Nb film has the largest *c* lattice constant, while PZT-Fe has the smallest one, with the un-doped PZT is in between.
- Coercive field for un-doped PZT is also in between PZT-Fe (lower coercive field) and PZT-Nb (higher coercive field), suggesting that in PZT-Nb the polarization is harder to switch than in PZT-Fe, a finding that can be also explained in relation with the magnitude of the leakage current. This result is contrary to the ones in the literature, claiming that acceptor doped PZT is hard while the donor doped PZT is soft^20,42^. In the three samples: when the leakage current is high the polarization switches faster, and the coercive field is lower because the compensation of the depolarization field is faster, which translates into a lower applied field needed to bring-in the necessary charges for compensation (one must not forget that the voltage for switching has a triangular shape, with linear dependence on time!); if the leakage is low then the time needed to bring-in the same amount of charge to compensate the depolarization filed will be larger, translated into a higher coercive field. Therefore, high leakage favours rapid switching, with low coercive field, while low leakage favours more gradual switching, with large coercive field. The coercive field can also be estimated from C-V characteristics presented in Fig. 3b) and PFM phase images presented in Fig. 4. Results and discussions are given in SM. One can only observe that the magnitude of the coercive field estimated from different methods can vary significantly, due to various factors: if there is an electrode in direct contact with the ferroelectric layer or the contact is just mechanic, as for PFM; the area of the contacts and the extension of the applied electric field outside the contact (stray field); the duration of the measurement, etc. It is not the scope of the present study to clarify these differences, and further studies are needed to decide on the best and trusted method to extract the coercive field. As resulting from the literature, hysteresis loop is the most used to extract this quantity.
- The internal electric field is larger in the doped samples, and it is in all cases orientated from the bottom SRO electrode towards the growing surface. However, its magnitude is about to 5 to 20 times lower than that of the coercive voltage, thus it cannot impose, alone, an upward direction of the polarization. The fact that this internal field has the same orientation in all films can be explained by the fact that all were grown on SRO/STO substrates, thus all are subject of the same strain/stress conditions, with negligible impact from the amount of doping as evidenced by the structural investigations (see the results of XRD and TEM investigations).
- Dielectric constants are slightly higher in the doped PZT films. This can be explained by the larger amount of structural defects carrying charges able to respond to the small a.c. voltage signal used for the C-V measurements (frequency of 100 kHz and amplitude of 200 mV).
- The height of potential barriers at electrodes are significantly different, again with un-doped PZT in-between PZT-Fe (lowest barrier) and PZT-Nb (highest barrier). The heights of the barriers, although small compared to the bandgap of PZT, are consistent with other previous reports in the literature^40,44,46,47^.
- The effective density of charge carriers (the difference between electrons and holes) is not significantly different in the three layers. The density of charge carriers is only very slightly larger in PZT-Fe, compared to un-doped PZT, while in the PZT-Nb is very slightly lower. This can be explained by the fact the Fe doping can promote formation of oxygen vacancies^24,48^, while Nb doping can inhibit formation of oxygen vacancies^42^.

The significant differences in the electric proprieties of the three samples do not correlate neither with the fraction of *a*-domains (and domain walls), nor with the amount of strained *c*-domains. Therefore, one can conclude that these differences are determined by the effect of doping on the properties of the preponderant (about 95%) "relaxed” PZT volume. However, the “strained” PZT layer located at the interface with the bottom SRO electrode may have some influence on the interface properties, leading to an asymmetry of the C-V and I-V characteristics, as observed in Fig. 3b,c (e.g. different peak capacitance values for PZT-Nb and PZT-Fe). In any case, the probable presence of asymmetric Schottky contacts at the electrode interfaces does not impact the procedures used to extract the relevant parameters of the interfaces, such as the height of the potential barriers or the effective density of free carriers. Similar procedures were used for semiconductor structures with asymmetric Schottky diodes or with strained metal–semiconductor interface^49–51^.

The electrical measurements do not help in deciding that PZT-Fe is p-type and PZT-Nb is n-type. The attempts to perform Hall measurements in order to establish the conduction type in PZT-Fe and PZT-Nb were not successful. In order to get some insight, PFM investigations were performed.

The reasoning is that un-doped PZT, which is assumed to be n-type due to the presence of oxygen vacancies acting as donor centres, grows with polarization upward, oriented from the bottom SRO contact towards the surface^24,48^. Therefore, one expects that PZT-Nb, assumed to be also n-type, grows also with upward polarization, while PZT-Fe, assumed to be p-type, may grow with downward polarization, considering that the sign of the carriers involved in the compensation of the depolarization field has changed from negative to positive. A special poling map was selected to investigate the polarization switching at nanoscale, as can be seen in Fig. 4a. The voltage applied on the PFM tip was linearly increased from − 10 to 10 V on each scan line, as in Fig. 4b.

The written ferroelectric domains can be observed in the piezoresponse phase images (see the inner square in Fig. 4d,f,h while the corresponding surface topography is shown in Fig. 4c,e,g. Analysing the phase images one can observe the followings:

- For as-grown un-doped PZT film the polarization is upward oriented (Fig. 4h). This is in agreement with previous reports^24,48^.
- For as-grown PZT-Nb (Fig. 4d) the dominant orientation of polarization is upward, but also there are some areas that seems to have downward polarization (these are estimated to about 5% of the as-grown area, see details in SM). The difference compared to un-doped PZT can be explained by a reduction of the density of oxygen vacancies, affecting the compensation of the depolarization field during the growth process. This result is in agreement with the fact that the leakage current is lower and the coercive field is larger in PZT-Nb.
- For PZT-Fe it appears to be a mixture of domains with upward and downward polarization, with larger area of downward polarization (estimated to about 82% of the as-grown area before poling the inner square in Fig. 4f, see figure SM12 in SM). This can be explained by the fact that Fe is an acceptor, favouring formation of oxygen vacancies for local charge compensation, thus one can expect that the amount of holes and electrons is not so drastically different. Depending on the distribution of Fe in the film and on the distribution of other structural defects, the charges available for the compensation of the depolarization field can favour upward or downward orientation of polarization. Nevertheless, the change of doping from Nb (donor) to Fe (acceptor) has a visible effect on the PFM phase signal.

**Figure 5:**
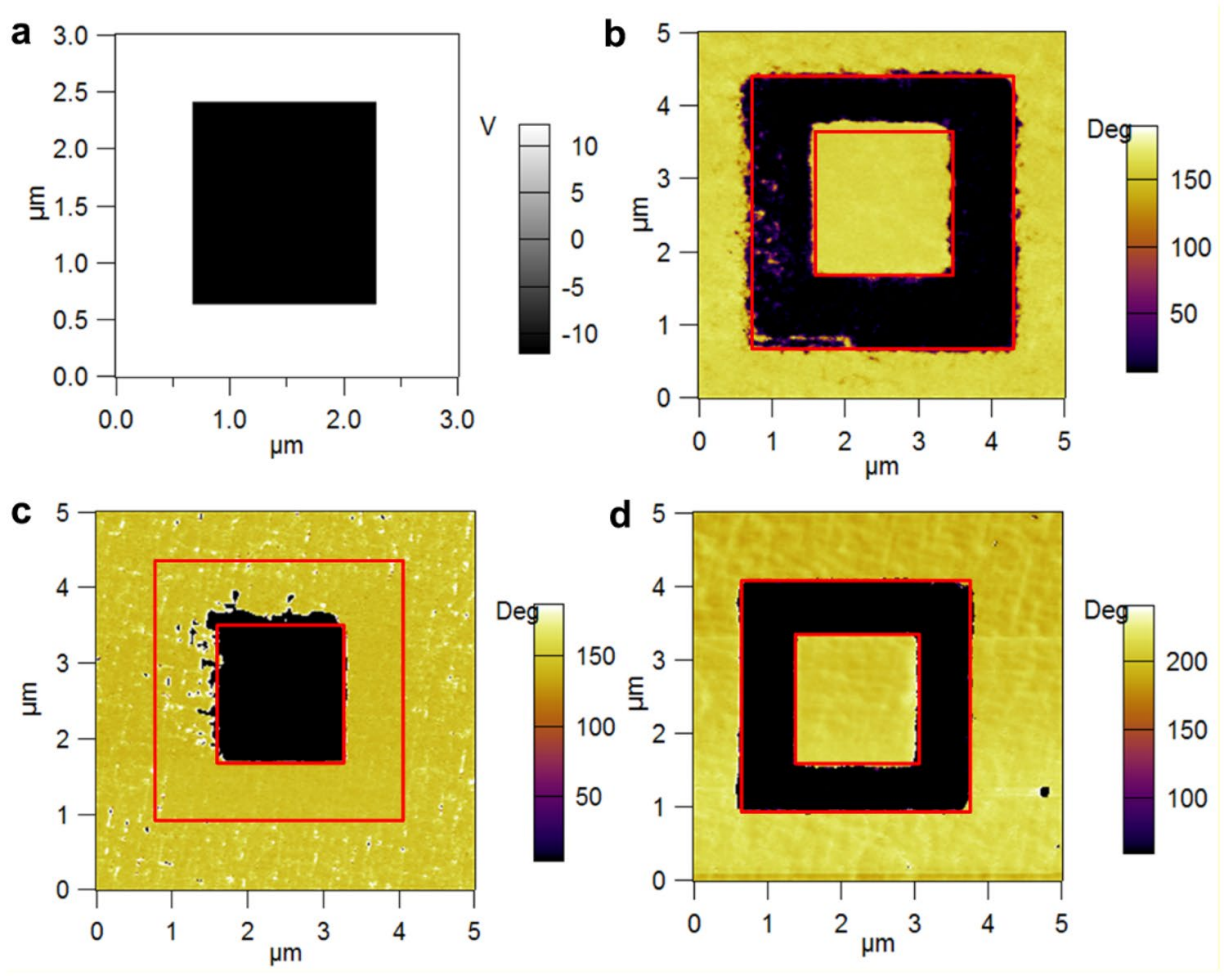
(**a**) Poling map applied for polarization switching; Piezoresponse phase images after domain writing according to the poling map on: (**b**) PZT-Nb; (**c**) PZT-Fe; (**d**) un-doped PZT thin films.

In order to clarity if the polarization orientation changes from upward in Nb-PZT (n-type doping) to downward in Fe-PZT (p-type doping), a box-in-box poling procedure was applied and the PFM phase images were recorded for the three samples (see Fig. 5). Analysing the poling PFM phase images one can observe that the PZT-Nb in the as-grown state (outside the boxes) has and upward polarization, the phase contrast being the same as in the inner box, which was poled by applying an external voltage of -10 V on the surface. On the other hand, looking at the as-grown surface of the PZT-Fe one can observe that this has a phase contrast similar to that of the outer box, which was poled by applying + 10 V on the tip, thus indicating a downward orientation of polarization for the as-grown sample. The un-doped sample shows similar behaviour with PZT-Nb, supporting intrinsic n-type doping due to oxygen vacancies. The results presented in Fig. 5 are strong evidences that polarization orientation can be controlled by changing the doping type in epitaxial PZT, in line with previous reports showing that polarization orientation can be manipulated by chemistry of the PZT layer or by the conduction type of the bottom electrode^31,32,37^.

**Figure 6:**
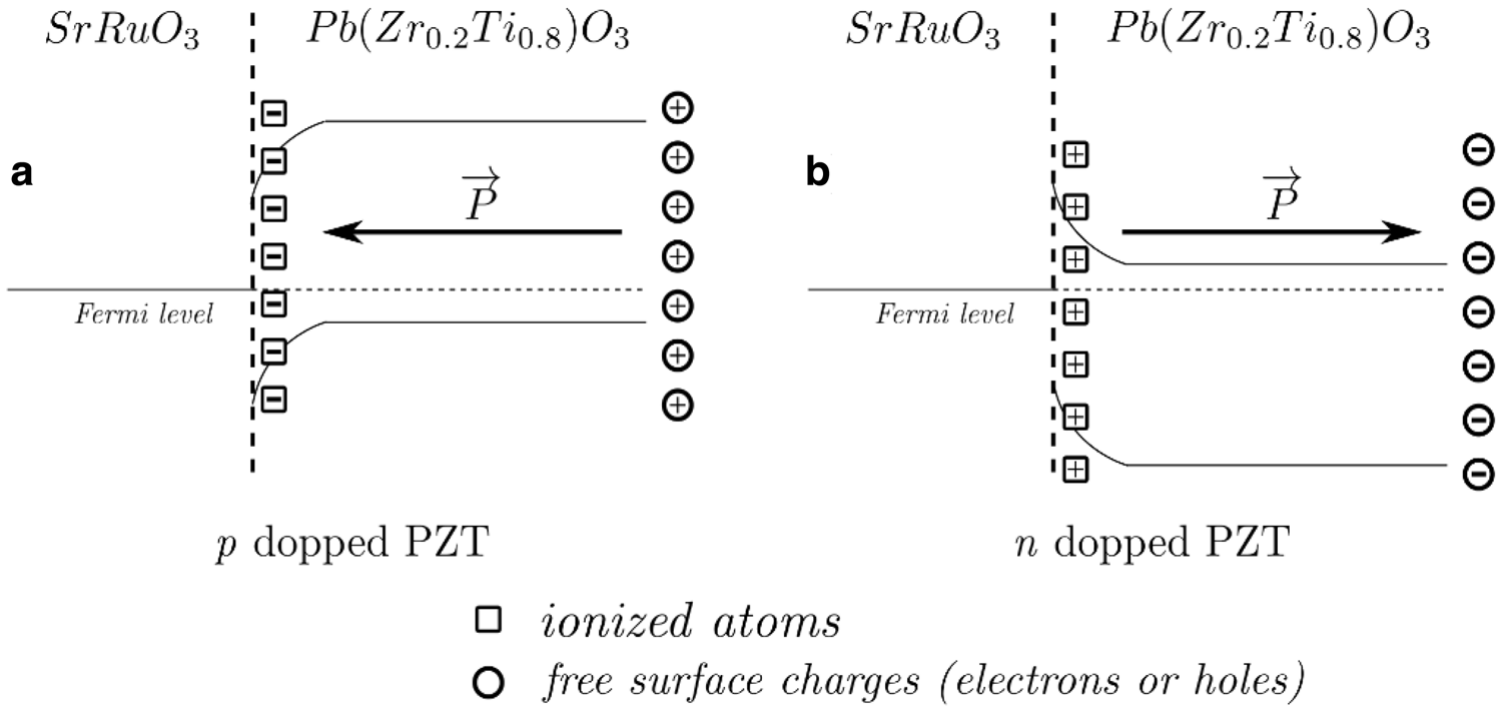
Band alignment sketch for the two doping cases, with the preferred polarization direction in the PZT. The squares mark the ionized atoms in the interface region that contribute to compensation of the polarization charges while the circles are the free surface charges.

The results presented in Figs. 4 and 5 can be explained as follows (in relation also to Fig. 6):

- Fe is introducing an acceptor level close to the valance band, as shown by first-principle calculations, while Nb is introducing a donor level close to the conduction band (details in SM); PZT-Nb is n-type and PZT-Fe is slightly p-type as suggested by the PFM poling results shown in Figs. 4d, f and 5b, c, respectively. The band diagrams for the two situations are schematically presented in Fig. 6a (p doping) and b) (n doping), together with polarization orientations in the as-grown samples and the sign of the compensation charges located near the interface with the bottom SRO electrode (ionized acceptors or donors) and the surface (free holes or electrons).
- The similar effective densities of free carriers correspond to the values of remnant polarization, confirming that polarization is controlling these densities either the PZT is un-doped or doped with acceptor Fe or donor Nb; the regulation of the necessary carrier densities to keep about the same polarization values is achieved by self-doping, most probably oxygen vacancies, considering that the contribution of free carriers from electrodes is similar, in all cases being used SRO electrodes.
- The difference in barrier height derives from the difference in the position of the Fermi level: for un-doped PZT the n-type character is due to oxygen vacancies, thus the Fermi level is close to conduction band; for PZT-Nb, the Fermi level position is dictated by the position of the donor levels introduced by oxygen vacancies and by Nb impurities, and by their degree of ionization; if one is completely ionized and the other is only partly ionized, then the Fermi level lays in between the two donor levels, a situation that can lead to a higher potential barrier compared to un-doped PZT; in PZT-Fe it appears that the Femi level is very close to valance band, leading to a low potential barrier.
- The differences in potential barriers reflect very well in the magnitude of the leakage current, which is about 2 orders of magnitude larger in PZT-Fe than in PZT-Nb, with un-doped PZT in between.

## Conclusions

In summary, a relatively low doping concentrations of Fe and Nb can trigger significant differences in the macroscopic properties of the epitaxial PZT films, although the structural properties are similar. The differences can be explained by different electronic properties induced by Fe doping, acting as acceptor, and Nb doping, acting as donors. The important result of the study is that the polarization orientation is different for n-type (upward) and p-type (downward) doping of the epitaxial PZT layers. Further studies are needed to confirm this finding and to conclude if polarization orientation can be directly linked to the doping type, thus to establish if a PZT layer is n-type or p-type doped only by analysing the polarization orientation in the as-grown samples. These results open new perspective in obtaining genuinely ferroelectric p-n homojunctions.

## Supplementary Information

Supplementary Information.
