# Author Correction: Controlling polarization direction in epitaxial Pb(Zr_0.2_Ti_0.8_)O_3_ films through Nb (n-type) and Fe (p-type) doping

**DOI:** 10.1038/s41598-022-13921-8

**Published:** 2022-06-01

**Authors:** Cristina Florentina Chirila, Viorica Stancu, Georgia Andra Boni, Iuliana Pasuk, Lucian Trupina, Lucian Dragos Filip, Cristian Radu, Ioana Pintilie, Lucian Pintilie

**Affiliations:** National Institute of Materials Physics, Atomistilor 405A, 077125 Magurele, Ilfov Romania

Correction to: *Scientific Reports* 10.1038/s41598-022-04802-1, published online 14 January 2022

The Acknowledgments section in the original version of this Article was incomplete.

“This research was funded by PCCF16/2018 (ID PN-III-P4-ID-PCCF-2016-0047) funded by the Ministry of Education and Research/UEFISCDI.”

now reads:

“This research was funded by PCCF16/2018 (ID PN-III-P4-ID-PCCF-2016-0047) funded by the Ministry of Education and Research/UEFISCDI. The fee for open access publication was supported from the project 35PFE/2021, funded by the Romanian Ministry of Research, Innovation and Digitization.”

The original Article has been corrected.

