# Supplementary material for: Controlling polarization direction in epitaxial Pb(Zr0.2Ti0.8)O3 films through Nb (n-type) and Fe (p-type) doping
Source: Sci Rep. 2022 Jan 14;12:755. doi: 10.1038/s41598-022-04802-1 (PMC8760319; doi:10.1038/s41598-022-04802-1)
Supplement: Supplementary file 1 — Supplementary Information. [file 41598_2022_4802_MOESM1_ESM.docx]

**Supplemental Material**

**Controlling polarization direction in epitaxial Pb(Zr_0.2_Ti_0.8_)O_3_ films through Nb (n-type) and Fe (p-type) doping**

Cristina Florentina Chirila^1^, Viorica Stancu^1^, Georgia Andra Boni^1^, Iuliana Pasuk^1^, Lucian

Trupina^1^, Lucian Dragos Filip^1^, Cristian Radu^1^, Ioana Pintilie^1^, and Lucian Pintilie^1,*^

^1^National Institute of Materials Physics, Atomistilor 405A, Magurele, Ilfov, 077125, Romania

*pintilie@infim.ro

**Targets preparation**

The un-doped target was prepared following the procedure described in *Nanomaterials* **11**, 1177 (2021). The doped targets were prepared following the solid-state reaction route. As mentioned in the main text, the doping was of 1%, with the following chemical formulae: PbZr_0.198_Ti_0.792_Fe_0.01_O_3_ (PZT-Fe) and PbZr_0.198_Ti_0.792_Nb_0.01_O_3_ (PZT-Nb). The starting precursors were: Pb oxide (PbO, 99.999%, Puratronic), Zr oxide (ZrO_2_, 99.99%, Aldrich), Ti oxide (TiO_2_, 99.99%, Aldrich), Fe oxide (Fe_2_O_3_ 99.7% Aldrich) and Nb oxide (Nb_2_O_5_, Aldrich 99.99%). Ethanol was used as binder during milling in a planetary mill from RETSCH, with a speed of 150 rpm for two hours. The powders were dried at 150 °C, followed by calcination at 850 °C for two hours. The calcination product was milled again for 3 hours, and then cold pressed in a stainless steel matrix of 40 mm diameter, using a pressure of 19 MPa. The sintering was performed at 1200 °C for two hous, with a heating rate of 2 °C/min. The ceramic disks were sintered in closed alumina recipients, surrounded by PbO powder to avoid Pb losses. The final density, estimated by Archimedes method is of 7.48 g/cm^3^. SEM images (see **Fig. SM1**) show compact ceramics with an average size of crystalline grains of about 4.22 μm for PZT-Fe and about 1.51 μm for PZT-Nb.

The XRD analyses were performed on polished surfaces of the targets, using CuK_α_ radiation in Bragg-Brentano measuring geometry. The measurements were performed within 2θ = 15 − 95^◦^, step size = 0.02^◦^, 1.5 s/step. The diffractograms were fitted by the Pawley method to determine the lattice parameters and the size of the coherence domains. Attempts were made to evaluate the structural changes induced by the doping atoms using the Rietveld method. The Pawley and Rietveld refinements were made with the TOPAS v.3 program (Bruker-AXS). The diffractograms are presented in **Fig. SM2** and the significant results are shown in **Table SM**1.

The lines are identified with PZT, without additional phases. The diffraction line positions of doped PZT are apparently the same as those of un-doped PZT and differ very little from the reference positions of PbZr_0.2_Ti_0.8_O_3_ from the ICDD database^1^, indicating that the size of the unit cell is very weakly affected. The significant differences in relative intensities indicate changes in the atomic structure of the unit cell and is evidence of the incorporation of the doping atoms in the PZT lattice. The lattice constants were precisely determined by Pawley whole pattern fitting, and attempts were made to estimate the probability of substituting the host atoms at A (Pb) or B (Ti or Zr) position by Rietveld refinement.

The modification of the lattice constants (**Table SM1** and **Fig. SM2b**) cannot be entirely understood considering only the differences between the atomic and ionic sizes of the host and doping atoms (listed in **Table SM2**). The increase of the lattice constant *a* at Nb doping could be a consequence of B site doping, as Nb(V) is larger than Ti(IV) which is preponderant at this site. The increase of *a* at Fe doping cannot be explained by ion sizes, as the Fe ions are smaller than all the host atoms. The considerable decrease of *c* at Fe doping could indicate that Fe substitutes Ti/Zr generating in the same time oxygen vacancies.


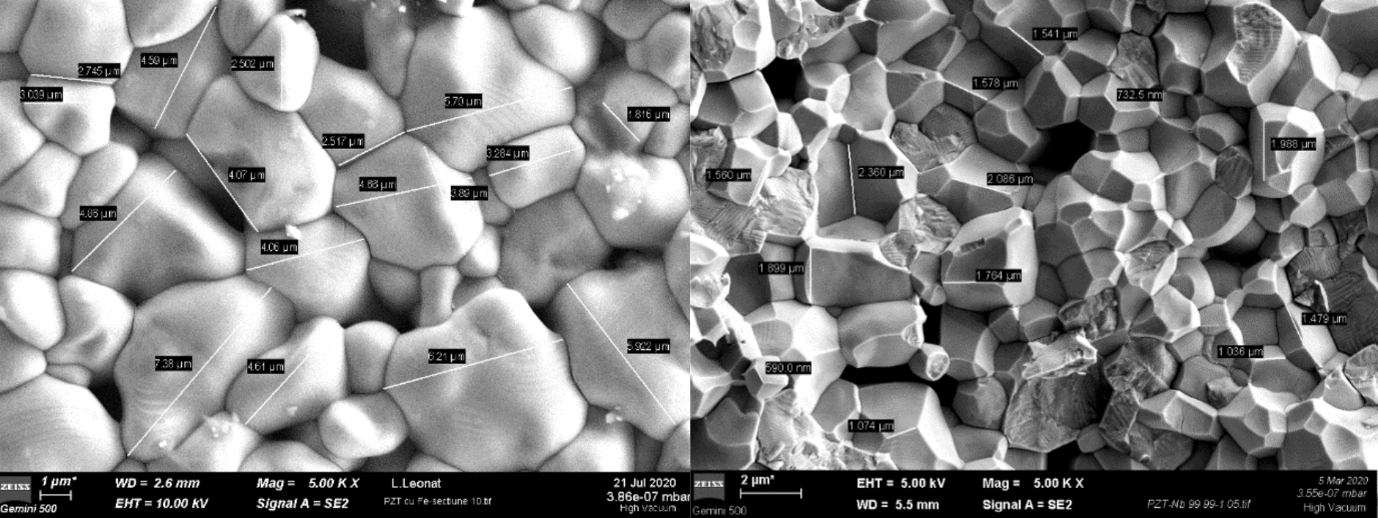


**Figure SM1** Cross section SEM images of PZT-Fe target (left) and PZT-Nb target (right).


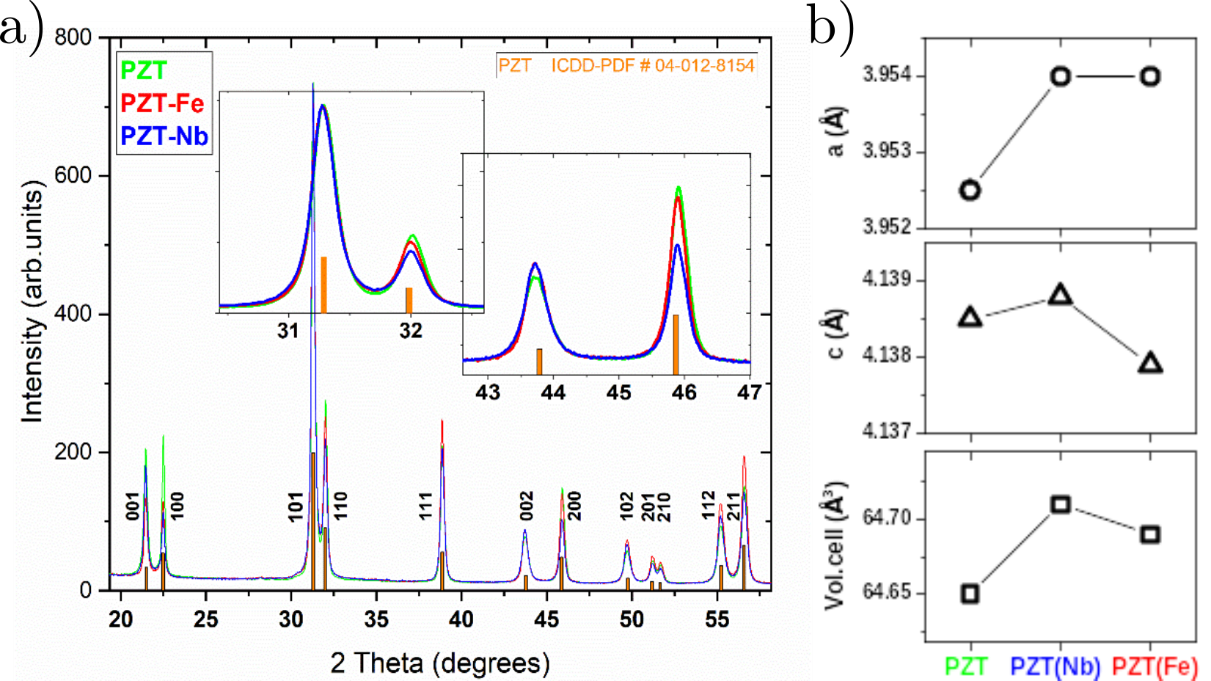


**Figure SM2** (a) Comparative representation of the XRD patterns of the pure, Fe-doped, and Nb-doped targets and the line indexing in the tetragonal system. The XRD lines of a reference PbZr_0.2_Ti_0.8_O_3_ from the ICDD database (ICDD # 04-012-8154) are also represented to compare the lines’ positions and relative intensities. The presented diagrams were obtained after applying to the original data the “sample-displacement” correction that resulted from the whole pattern fitting using the TOPAS software. Additionally, the patterns were normed at the intensity of the most intense line, 101. (b) The lattice constants and the unit cell volume determined by Pawley whole pattern fitting.

**Table SM1 Lattice constants**

| Sample | a (Å) | c (Å) | c/a | V_cell_ (Å^3^) | Domain size (nm) |
| --- | --- | --- | --- | --- | --- |
| PbZr_0.2_Ti_0.8_O_3_  ICDD PDF # 04-012-8154 | 3.9539 | 4.1319 | 1.045 | 64.59 |  |
| Target PZT | 3.9525 | 4.1385 | 1.047 | 64.65 | 48 |
| Target PZT(Nb) | 3.9540 | 4.1388 | 1.047 | 64.71 | 36 |
| Target PZT(Fe) | 3.9540 | 4.1379 | 1.047 | 64.69 | 42 |

**Table SM2.** Atomic and ionic sizes (in picometers)^2^

| _Z_Element | _82_Pb | _22_Ti | _40_Zr | _41_Nb | _26_Fe |
| --- | --- | --- | --- | --- | --- |
| [Atomic radius (empirical)](https://www.webelements.com/periodicity/atom_radius_emp/) | 180 | 140 | 155 | 145 | 140 |
| Ionic size in octahedral coordination  (Ionization state) |  | 74.5  (IV) | 86  (IV) | 78  (V) | 75 / 69  (II / III) |

**Deposition parameters**

The surface of the SrTiO_3_ (STO) substrate with (001) orientation was cleaned in hydrofluoric acid (HF) buffer solution and then annealed in air at 1000 °C for 2h. Smooth terraces of one-unit cell step were obtained in this way. A SrRuO_3_ (SRO) epitaxial buffer layer with a thickness of around 20 nm has been deposited on STO, this acting as a bottom electrode and also as an excellent template for the heteroepitaxial growth of high-quality ferroelectric perovskites. The bottom electrode was deposited at a substrate temperature of 700 °C, in a background atmosphere of 0.133 mbar oxygen, with a laser fluence of 2 J/cm2 and a repetition rate of 5 Hz. The PZT thin films were deposited at 575 °C, 0.2 mbar oxygen atmosphere, with a laser fluence of 2 J/cm2 and a repetition rate of 5 Hz. After deposition, the PZT layers were annealed at 575 °C in oxygen atmosphere at 1 bar, for 1 h, to compensate the oxygen vacancies.

**Quantitative analysis of the PZT phase composition.**

The relative amount of the strained *c*-domains of PZT and of the PZT *a*-domains were estimated by determining the intensities of the corresponding nodes in the reciprocal space mappings (RSM) in the vicinity of the -103 STO node, as shown in **Fig. SM3**.


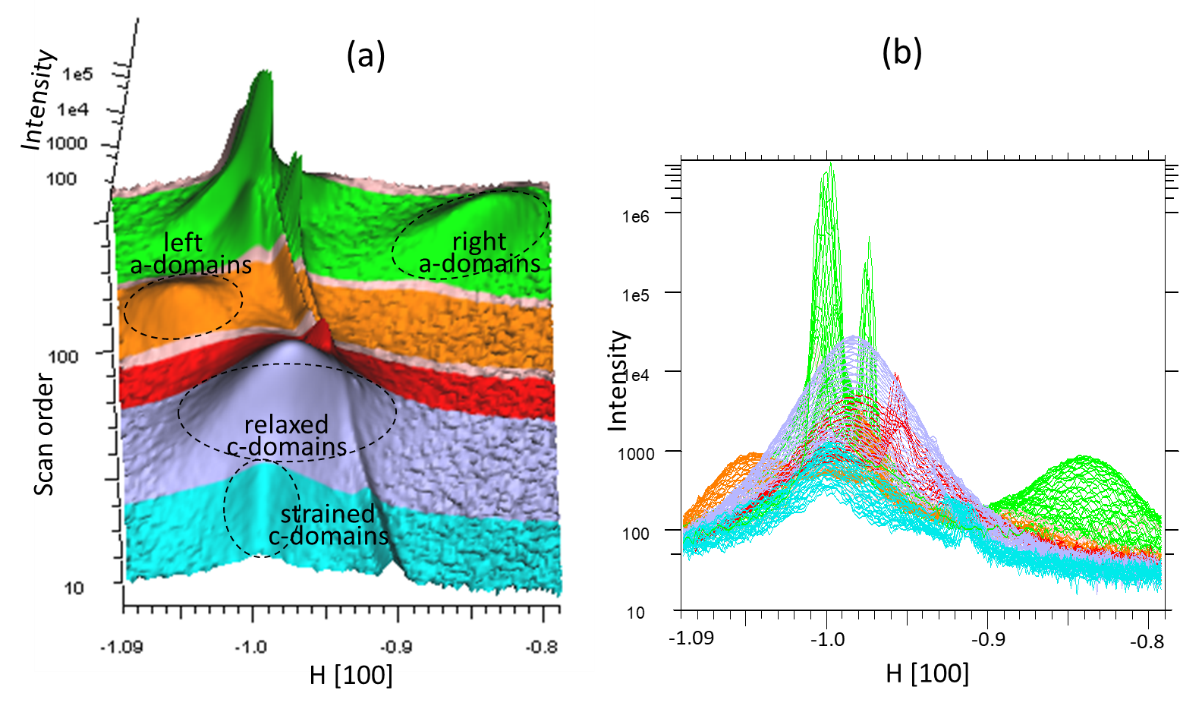


**Figure SM3**. The peaks used for the quantitative estimation of the PZT phase components, exemplified on the data of the PZT-Nb sample; left and right *a*-domains refer to the *a*-domains symmetrically inclined to left and right from the film surface. (a) 3D representation; (b) 2D representation used for the peaks’ area determination.

The results are presented in **Table SM3**.

**Table SM3**. Estimates of the PZT phase contents, based on the RSM peak intensities.

| Sample | a-domains / whole PZT  (%) | strained c-domains /whole c-domain PZT  (%) |
| --- | --- | --- |
| PZT | 7 | <1 |
| PZT-Nb | 3 | 3.5 |
| PZT-Fe | 3 | 5.5 |

**Electrical measurements**

**Determination of *N_eff_***

The fallowing deduction are performed considering that ferroelectric PZT is a wide gap semiconductor and the ferroelectric-metal contacts are Schottky-type contacts. Thus, metal-ferroelectric-metal (MFM) structures can be considered as two back-to-back connected Schottky diodes. One of the diodes will be reversed biased whatever the polarity of the applied voltage and in this way the capacitance of the MFM structure can be defined by (for voltage ranges where the polarization in totally reversed and saturted)^3^:

| $\frac{C}{A}=\frac{\varepsilon_{0}\varepsilon_{st}}{w}=\sqrt{\frac{q\varepsilon_{0}\varepsilon_{st}N_{eff}}{2\left( V+V_{bi}^{'} \right)}}$ | (1) |
| --- | --- |

where, A is the geometrical area defined by the top metallic contact, ε_0_ is the permittivity of the free space, ε_st_ is the static dielectric constant, q is the elementary charge, N_eff_ is the effective charge density, V is the applied voltage, *w* is the width of the depletion layer, and $V_{bi}^{'}$ is the apparent builtin potential.

The N_eff_ values are determined by the derivative of the $\frac{1}{C^{2}}=f\left( V \right)$ representation:

| $N_{eff}=\frac{2}{q\varepsilon_{0}\varepsilon_{st}\left[ \frac{d1/{C^{2}}}{dV} \right]}$. | (2) |
| --- | --- |

**Determination of the potential barrier**

The dominant conduction mechanism is considered to be the thermionic emission with the mean free path of the charge carriers in PZT much smaller than the film thickness, thus the current density, J, is described by the Schottky–Simmons equation^4^ (the injection is limited by the potential barriers at the electrode interfaces and the drift–diffusion in the film is limited by the carriers mobility)^1, 5, 6^:

| $J=2q\left( \frac{2\pi m_{eff}kT}{h^{2}} \right)^{\frac{3}{2}}\mu Ee^{-\frac{q}{kT}\left( \phi_{B}^{0}-\sqrt{\frac{qE_{m}}{4\pi\varepsilon_{0}\varepsilon_{op}}} \right)}$ | (3) |
| --- | --- |

where, h is the Planck’s constant, m_eff_ is the effective mass, μ is the mobility, E is the applied electrical field, k is the Boltzmann’s constant, ε_op_ is the optical dielectric constant, ϕ^0^_B_ is the interfacial potential barrier height at zero volts, E_m_ is the maximum electric field at the interface of the Schottky contact, and T is the temperature.

From the slope of the Arrhenius plot $ln\left( J/{T^{\frac{3}{2}}} \right)=f\left( {1000}/T \right)$ as it is represented in **Fig. SM7**-middle line) for un-doped PZT poled with -6V at room temperature, the apparent potential barrier is obtained for different applied voltages, $\phi_{B}=\phi_{B}^{0}-\sqrt{\frac{qE_{m}}{4\pi\varepsilon_{0}\varepsilon_{op}}}$. Then, $\phi_{B}^{0}$ is evaluated from the intercept of the linear dependence of the $\phi_{B}=f\left( \sqrt{V} \right)$ as is presented in **Fig. SM7**-lower line.

In the following graphs (**Fig. SM4-SM9**) are given examples on how the values of N_eff_ and of the potential barriers at electrode interfaces were estimated for un-doped PZT, PZT-Nb and PZT-Fe using the above described procedures.

One can observe that the barrier for PZT-Nb is higher for high voltages applied on the sample, probably due to the fact that the Fermi level changes its position in the band gap.


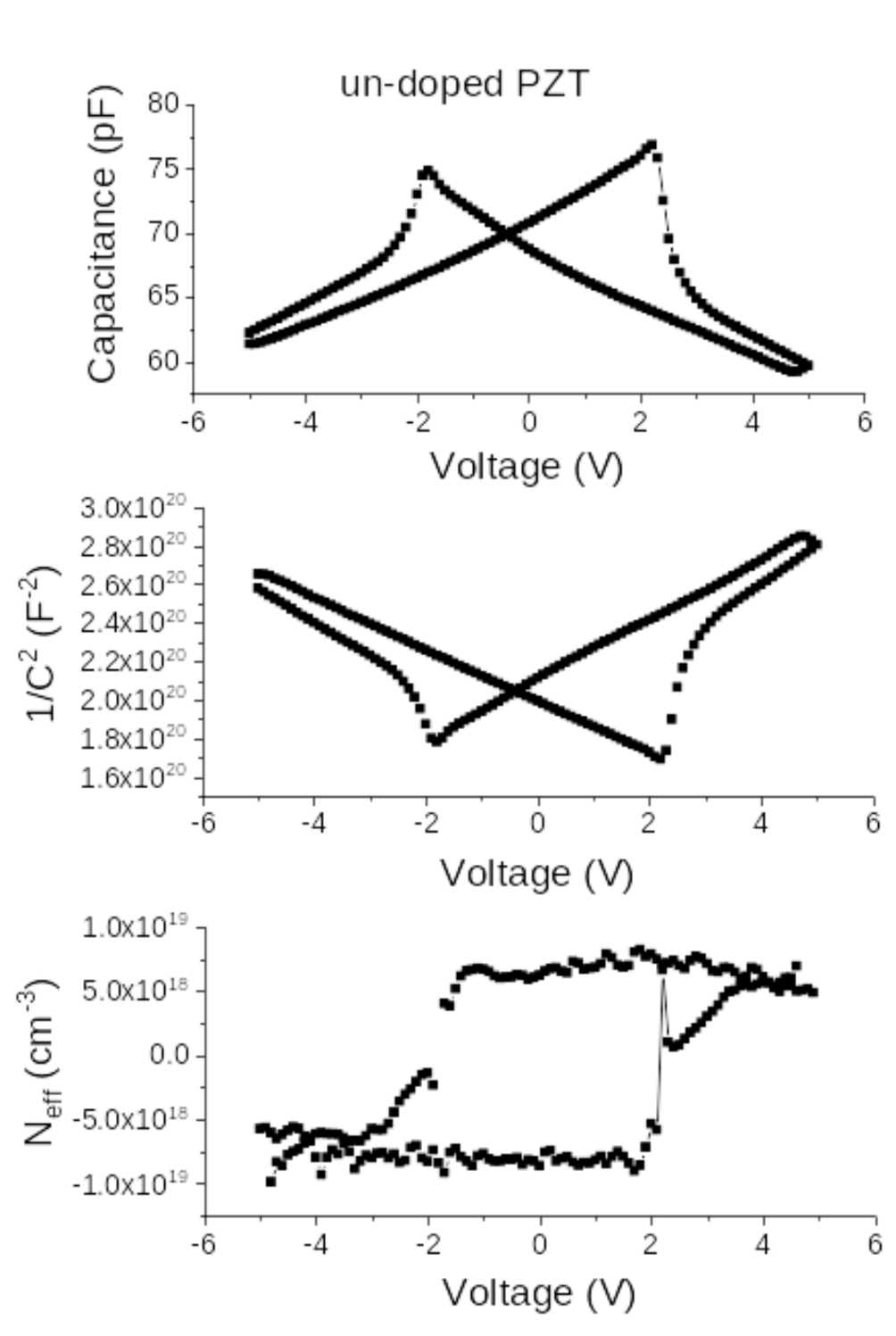


**Figure SM4** C-V characteristic (upper line), 1\C^2^ (middle line) and N_eff_ calculated using equation (2) (lower line) for un-doped PZT.


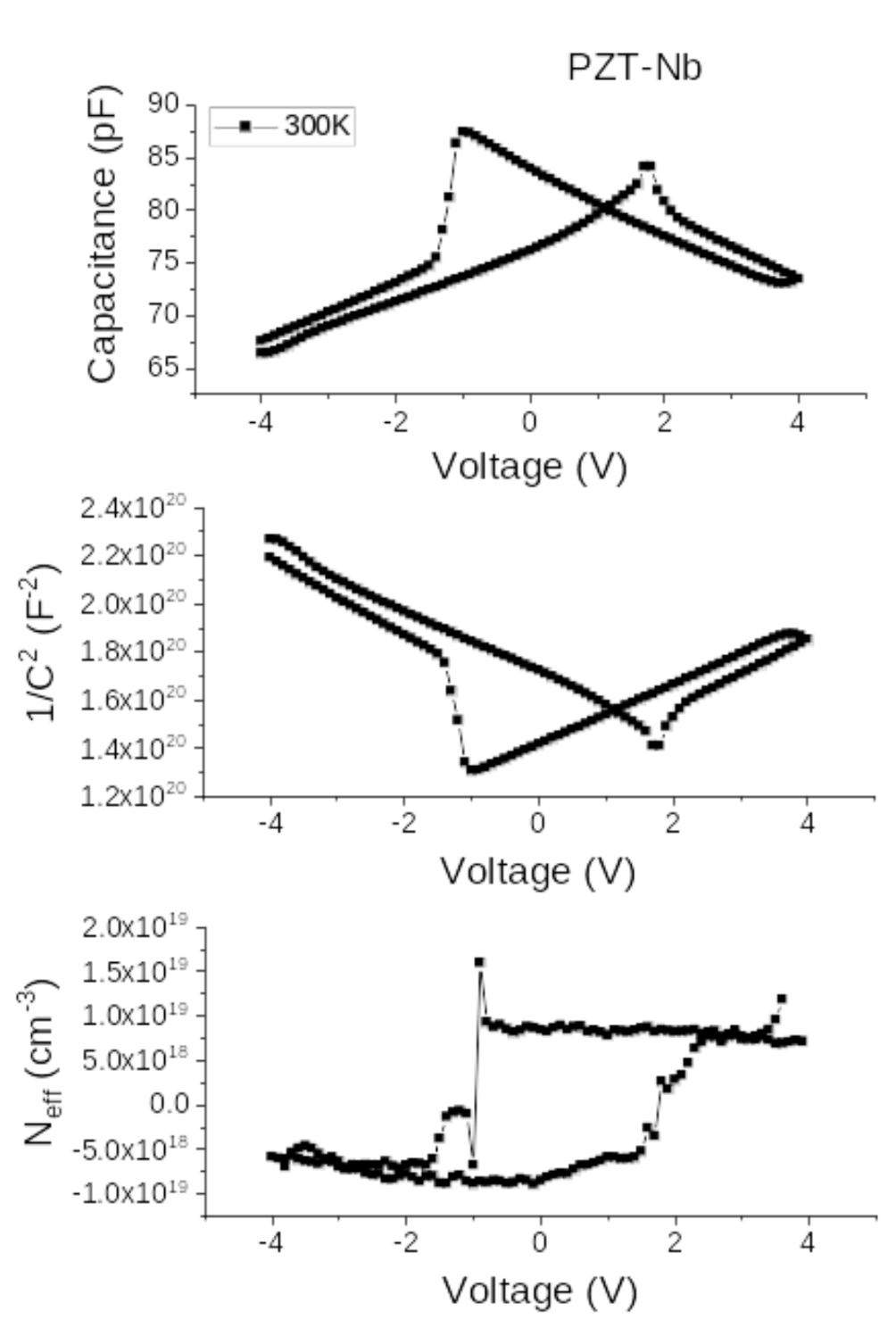


**Figure SM5** C-V characteristic (upper line), 1\C^2^ (middle line) and N_eff_ calculated using equation (2) (lower line) for PZT-Nb.


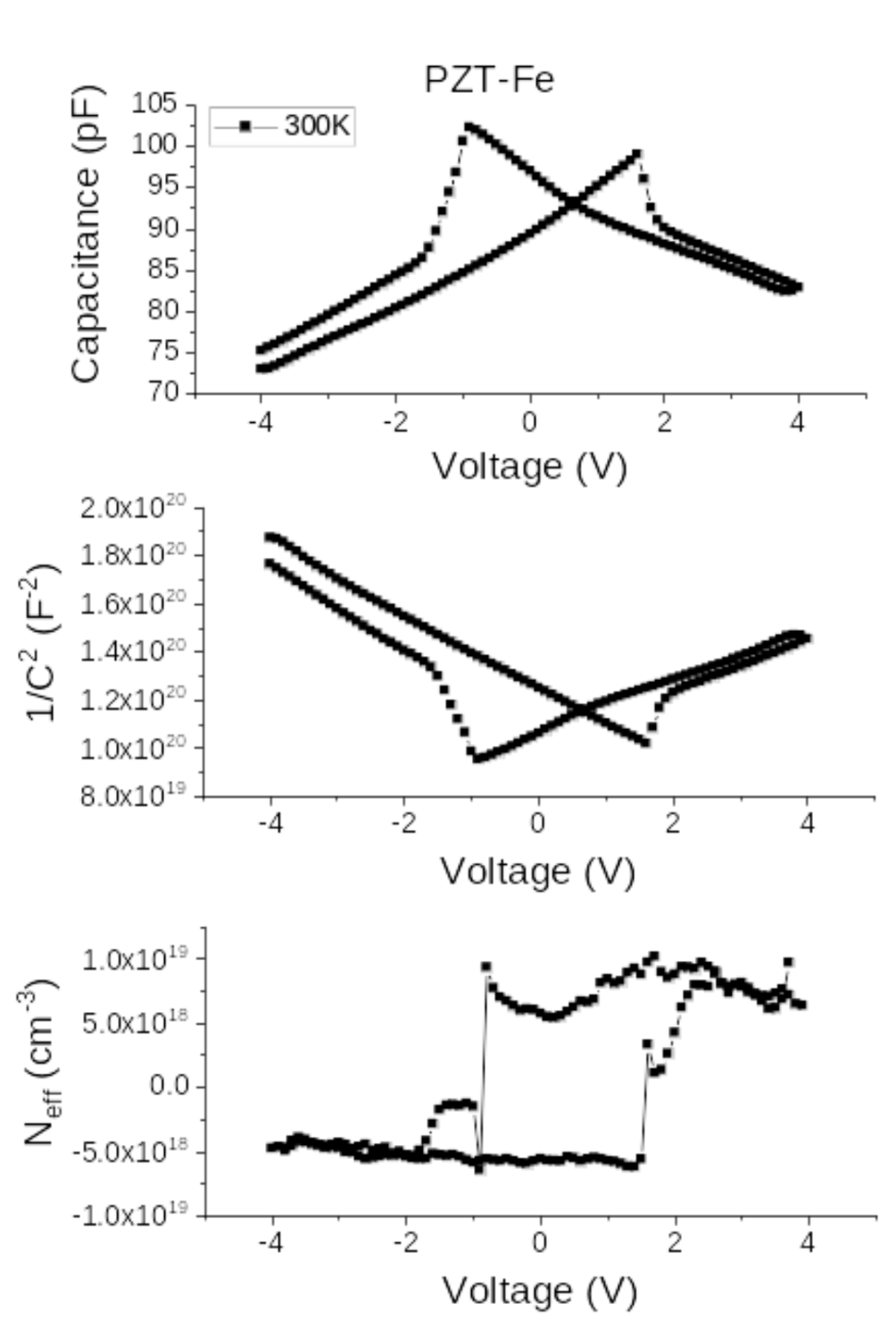


**Figure SM6** C-V characteristic (upper line), 1\C^2^ (middle line) and N_eff_ calculated using equation (2) (lower line) for PZT-Fe.


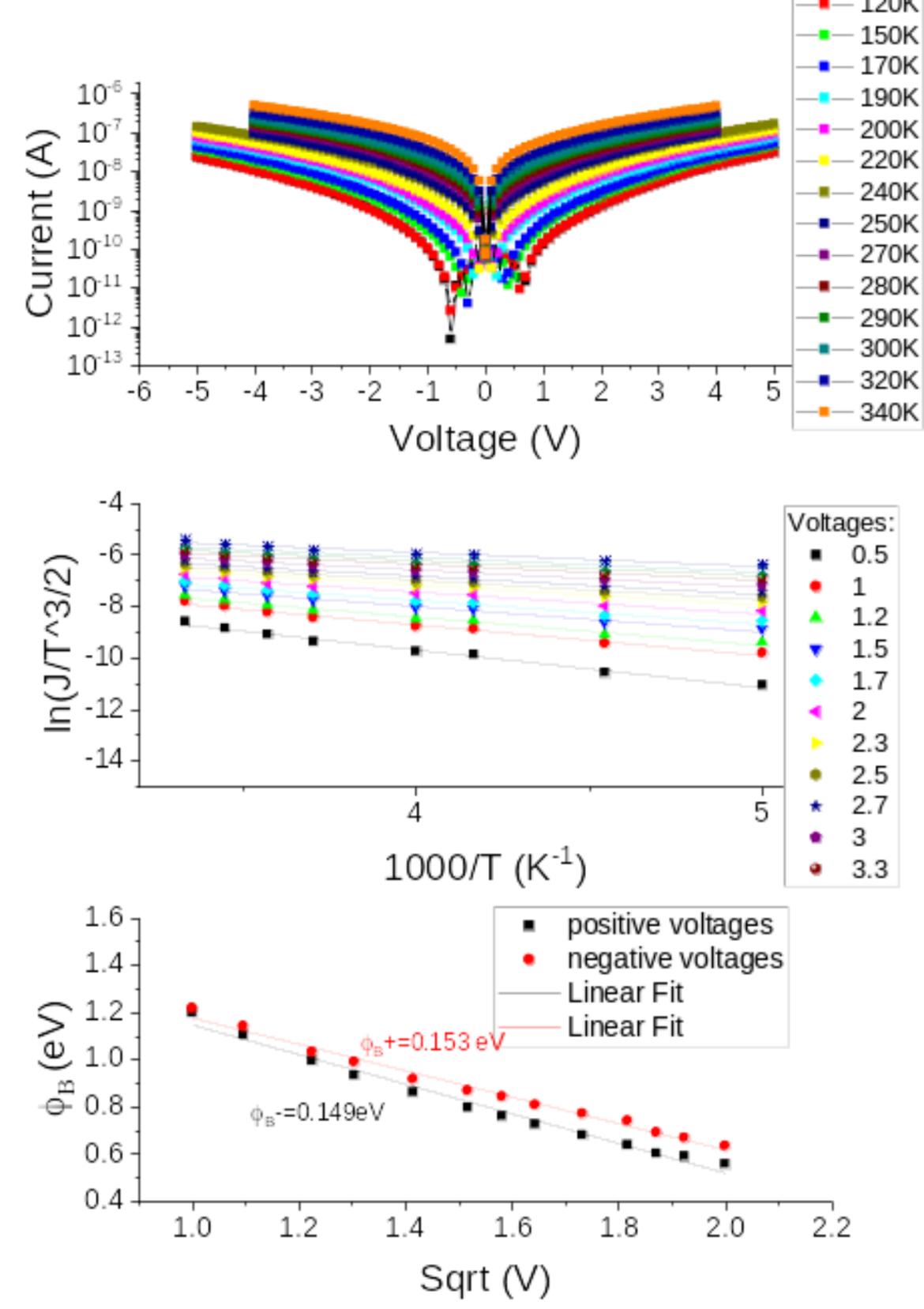


**Figure SM7** I-V characteristic (upper line), ln(J/T^3/2^)~1000\T representation (middle line) and apparent potential barrier as function of V^1/2^ (lower line) for un-doped PZT.


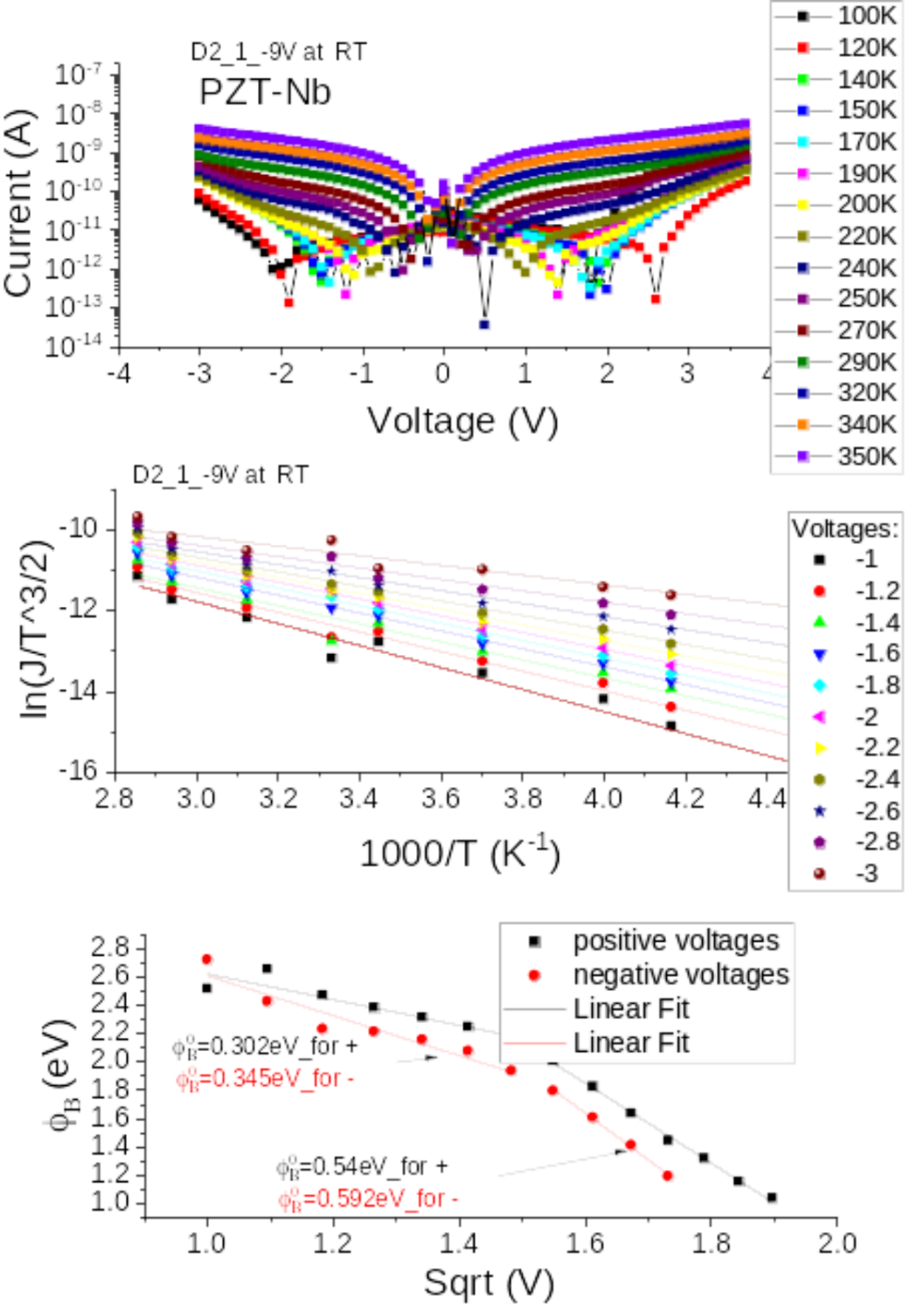


**Figure SM8** I-V characteristic (upper line), ln(J/T^3/2^)~1000\T representation (middle line) and apparent potential barrier as function of V^1/2^ (lower line) for PZT-Nb.


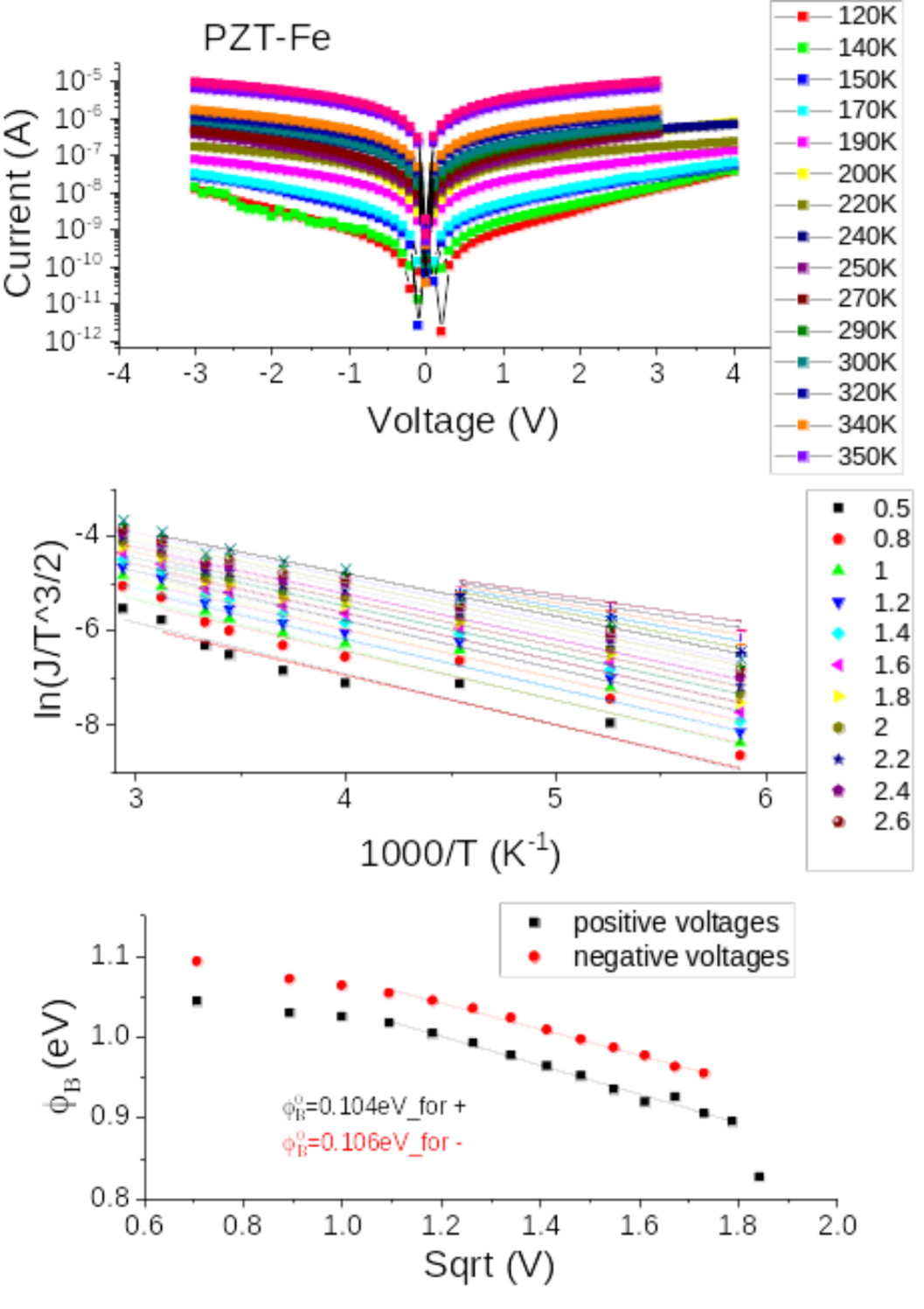


**Figure SM9** I-V characteristic (upper line), ln(J/T^3/2^)~1000\T representation (middle line) and apparent potential barrier as function of V^1/2^ (lower line) for PZT-Fe.

**Errors in estimating of the values presented in Table 1 in the main text and in SM**

The measurements of capacitance and polarization have high accuracy. The errors in the estimation of the coercive electric field and dielectric constant are mostly related to the accuracy of thickness and electrode area measurements. The thickness error is around 1-5 nm (maximum 3 %) depending on the sample, while the error in electrode area is estimated to be below 1 % (maximum 100 nm on each side of the rectangular mask, due to a possible shadow effect during electrode deposition). Thus, the errors for polarization, coercive field and dielectric constant are no larger than 3 %. The fitting errors for potential barrier are 0.0025eV, 0.009eV and 0.02eV for un-doped PZT, PZT-Fe and PZT-Nb, respectively. The errors were included in Table 1 in the revised version.

N_eff_ estimation seems to have many errors sources. Besides the errors related to thickness and electrode area measurements, another source of errors can be the value of the dielectric constant used for estimating N_eff_, knowing that the dielectric constant is both voltage and frequency dependent at room temperature. We have used the values estimated from the C-V characteristics recorded at 100 kHz, with capacitance at maximum applied voltage, considering that polarization contribution is minimized because the polarization is already saturated).

**I-V characteristics**

The presented current-voltage (I-V) measurements are performed after a total poling of samples with +V_max_ or –V_max_ and then returning the voltage to zero. The total measured current has two components: $I=I_{leakage}+I_{displacement}$. The displacement current is given by $I_{displacement}=\frac{CdV}{dt}$. The voltage is decreased from a maximum value towards zero, thus $I_{displacement}$ has an opposite sign to the $I_{leakage}$. When the displacement current is larger than the leakage current, then the measured current is negative. However, we have used the absolute values of the measured currents to represent I-V characteristics in logarithmic scale. This is the reason why those current minima occur in the I-V characteristics. An example is given in **Fig. SM10**, on how it looks the recorded I-V characteristic in the case of PZT-Nb, and how it looks after transformation into logarithmic scale using the absolute values of the current.


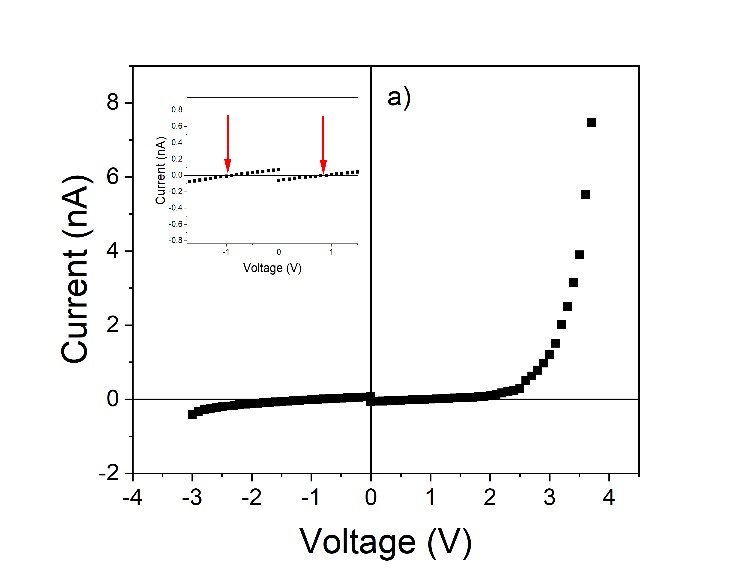

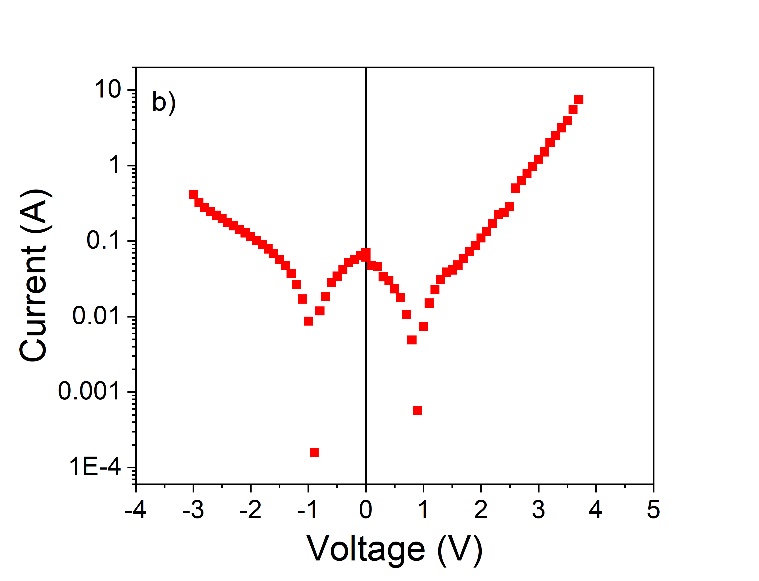


**Figure SM10** a) the as-recorded I-V characteristic at room temperature for PZT-Nb capacitor; the inset is a magnification of the characteristic around origin, where the total current has opposite sign to the applied voltage, and the red arrows point the voltages where current “minima” occurs when representing the absolute values of the current in logarithmic scale, as shown in b).

**Estimation of the coercive fields from C-V and PFM measurements**

The coercive fields were estimated from the voltages corresponding to maximum capacitance values in the C-V characteristics presented in Fig.3 in the main text. The average between the negative and positive coercive fields are presented in **Table SM4**.

The coercive field was also estimated from the PFM phase images, using the voltage corresponding to 50 % change in the phase, as exemplified in **Fig. SM11** for the un-doped PZT. The results for all samples are presented in **Table SM4**, together with the coercive field obtained from the hysteresis loops (see also Table 1 in the main text).


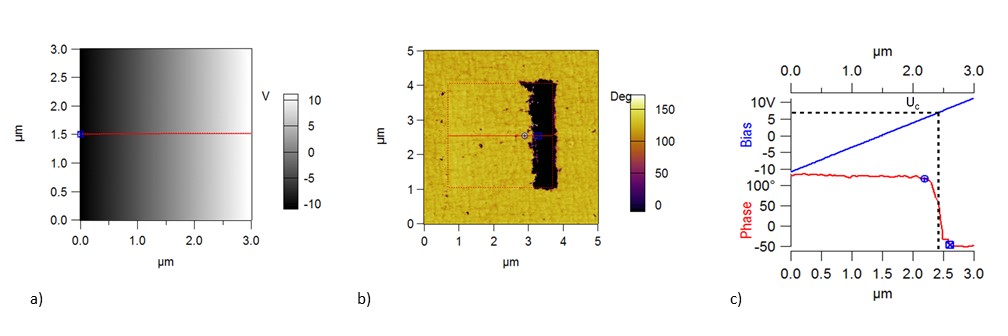


**Figure SM11** The poling map a); the phase image b); the voltage and phase spatial variation along the red line in a) and b). The dotted lines in c) mark the point where the phase has changed with 50 % and the corresponding voltage, which was then considered to estimate the coercive field.

**Table SM4** The values of the coercive fields estimated from hysteresis loops, C-V characteristics and PFM phase images.

|  | PZT | PZT-Nb | PZT-Fe |
| --- | --- | --- | --- |
| E_C_ from hysteresis loops | 247 | 338 | 130 |
| E_C_ from C-V characteristics | 117 | 83 | 70 |
| E_C_ from PFM phase images | 400 | 296 | 288 |

We do not comment on the magnitude of the coercive fields extracted from different measurements, just observe that the values extracted from the hysteresis loops are larger than the ones extracted from the C-V characteristics, while those extracted from PFM are considerably larger than those extracted from hysteresis and C-V measurements. It is out of the scope of the present study to explain these differences, as it may require more extended experiments.

We also mention that Piezoresponse force microscopy (PFM) is mainly a qualitative investigation method, giving an image of domain structure. It is also used to check the presence of ferroelectricity by upward and downward poling of specific areas of the samples. The voltage applied to the cantilever does not always represent the voltage drop on the sample because the surface of the sample or of the cantilever tip may be contaminated, requiring higher voltages to switch the polarization. Therefore, is preferable to avoid a quantitative evaluation of the results.

**Estimation of the areas with upward and downward polarization from PFM measurements**

The acquired data was processed with IGOR pro software embedded in the MFP 3D atomic force microscope. In the phase images of the piezoresponse signal, the color variation representing ferroelectric domains with opposite polarization was marked by means of a red mask. Piezoresponse phase images of PZT-Nb and PZT-Fe thin films with red mask applied are shown in **Fig. SM12** a) and b). The particle analysis was done only on the surface outside the blue square in order to determine the percentage of domains with polarization facing up or down in as-grown PZT-Nb and PZT-Fe layers, respectively. The calculated value for the area occupied by downward-facing domains in the as-grown PZT-Nb thin film is about 5% while for the as-grown PZT-Fe the area occupied by upward-facing domains is about 18%.

It should be noted that the values obtained are approximate because they are strongly influenced by the cantilever tip size (lateral resolution of the AFM) which means that there is not always an abrupt transition between adjacent domains and their delimitation from each other. The error was estimated to +/ − 3%.


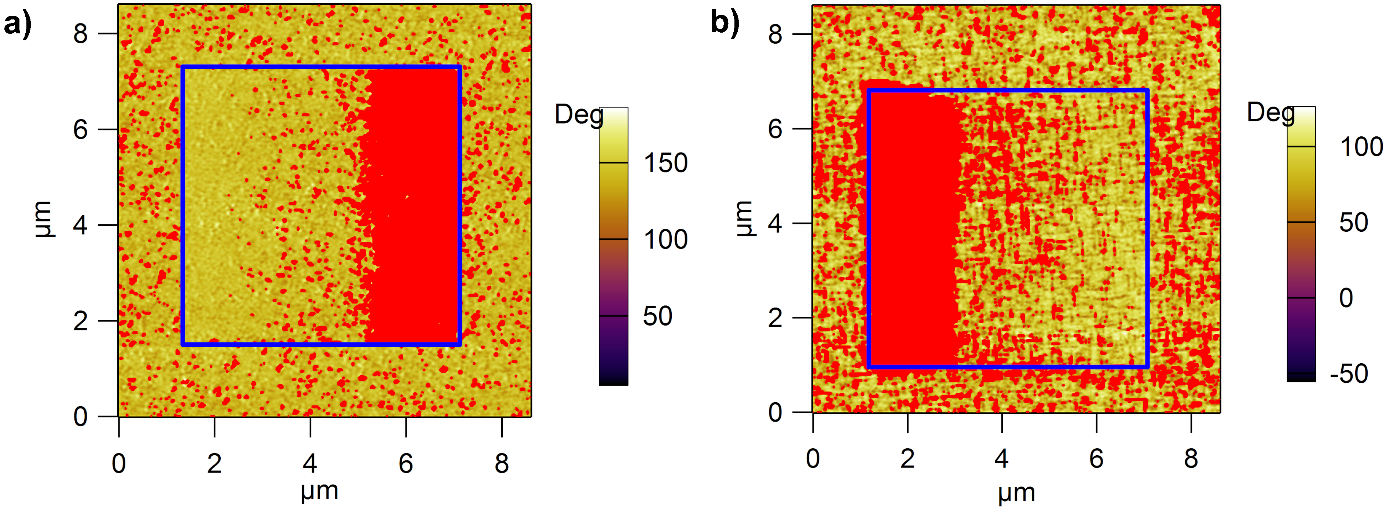


**Figure SM12** Piezoresponse phase images of PZT-Nb (a) and PZT-Fe (b) thin films with red mask applied showing the ferroelectric domains.

**2 Summary of DFT calculations**

**Calculation details**

The calculations were performed within the generalized gradient approximation (GGA) using the Quantum ESPRESSO plane-wave code^7^ , and the exchange correlation functional in the parametrization for solids (PBEsol)^8, 9^. We used Optimized Norm-Conserving Vanderbilt pseudo-potentials from the PseudoDojo library^10^.

A supercell geometry was used for all calculations. It was constructed starting from the tetragonal PbTiO_3_ (PTO) unit-cell that was constrained in the (xOy) plane to the SrRuO_3_ (SRO) lattice constant and then a vc-relax (variable cell relaxation) calculation was performed and the new unit-cell dimensions were: *a* = 3.905 Å and *c* = 4.092 Å with c/a = 1.047, which is in reasonable agreement with experimental and numerical data in the literature.

Using the optimized coordinates and lattice constants, a 3 × 3 × 3 supercell was constructed and two doping atoms (Fe and Nb) substituted the center Ti atom in the supercell. The result was a doping concentration of roughly 3% in the PbTiO_3_ bulk. The internal coordinates of the three super-cells were then relaxed until the Hellman-Forces were converged to 10^−5^ Ry/bohr with a kinetic energy cutoff of 70 Ry on a uniform Monhorst-Pack^11^ 4 × 4 × 4 grid. After the internal coordinates were relaxed, the defect formation energies and the density of states were computed for the pristine and doped structures. Furthermore, an interface was then constructed between cubic and relaxed (with and without doping) and the density of states at this interface was investigated.

**Results**

The defect formation energies per atom for the Fe and Nb doping were obtained using the following formula:

| $E_{formation}=E_{doped}-E_{pristine}-E_{X}+E_{Y}$, | (4) |
| --- | --- |

where, $E_{formation}$ is the formation energy of doping with species X, $E_{doped}$ is the total energy of the doped structure, $E_{pristine}$ is the total energy of the pristine structure, $E_{X}$ is the total energy of the doping atom placed in a box with the dimensions of the considered supercell and similarly $E_{Y}$ is the total energy of the atom that is replaced by species X (in this particular case, the Ti atom for all calculations). From Eq. 4 the formation energy for Fe and Nb doping are 0.0375 eV/atom and -0.0118 eV/atom, respectively. Ideally, the formation energy must be negative in order for a given structure to be stable. However, for values closer to zero it is possible that a metastable state can be reached and in this case the Fe doping may be possible. It should be noted that Fe doping is not a simple substitution of a Ti atom and usually it is accompanied by O vacancies which could account for the positive value of the formation energy^12^.

The density of states for the bulk PTO with and without doping are shown in **Fig. SM13**.

It can be observed that the Fe doping (bottom panel in **Fig. SM13**) introduces some states above the valence band maximum when compared with the pristine PTO case suggesting that it may behave as a p-type donor. The Nb doping (top panel) introduces some states in the conduction band of PTO but it is not clear if it acts as an n-type donor.

Using the relaxed internal coordinates for the above bulk devices, three heterostructures were constructed using a 3 × 3 × 3 SRO super-cell and the corresponding super-cells for the PTO cases. The resulting structure contains 270 atoms with the bottom SRO section fixed to an unrelaxed cubic symmetry and for the top PTO atoms, the bulk relaxed coordinates were used. A full relaxation of the structure would have taken a very long time. Also, previous studies of the SRO/PTO interface have shown that for three SRO unit-cell thickness in the direction perpendicular to the interface, there is a strong influence of the PTO polarization and the interface is strongly disturbed. SRO regains its bulk like properties for thicknesses in excess of 7 unit cells. For this case however, it is impossible to create such a thickness for SRO. Performing a simple self-consistent calculation for the SRO/PTO structures we can obtain the density of states for the three cases. It is very useful to plot the density of states (DOS) in a layer by layer fashion. **Fig. SM14** shows the layer by layer DOS per unit cell for the SRO/pristine-PTO interface. The energy is aligned at the Fermi level of the structure. Unfortunately, this preliminary result exhibits a known problem of treating ferroelectric-metal interfaces within DFT^13^. The band gap of PTO falls below the Fermi level in the SRO electrode which contradicts the experimental reality. The interface distortion is present even in the Fe doped PTO structure in **Fig. SM15**. The Fermi level intersects the conduction band of the PTO in all three layers. This trend is maintained for the Nb doped case, see **Fig. SM16**. The density of states of the two doping atoms appear on either side of the Fermi level, however the overall PTO DOS is shifted such that the conduction band intersects the Fermi level.


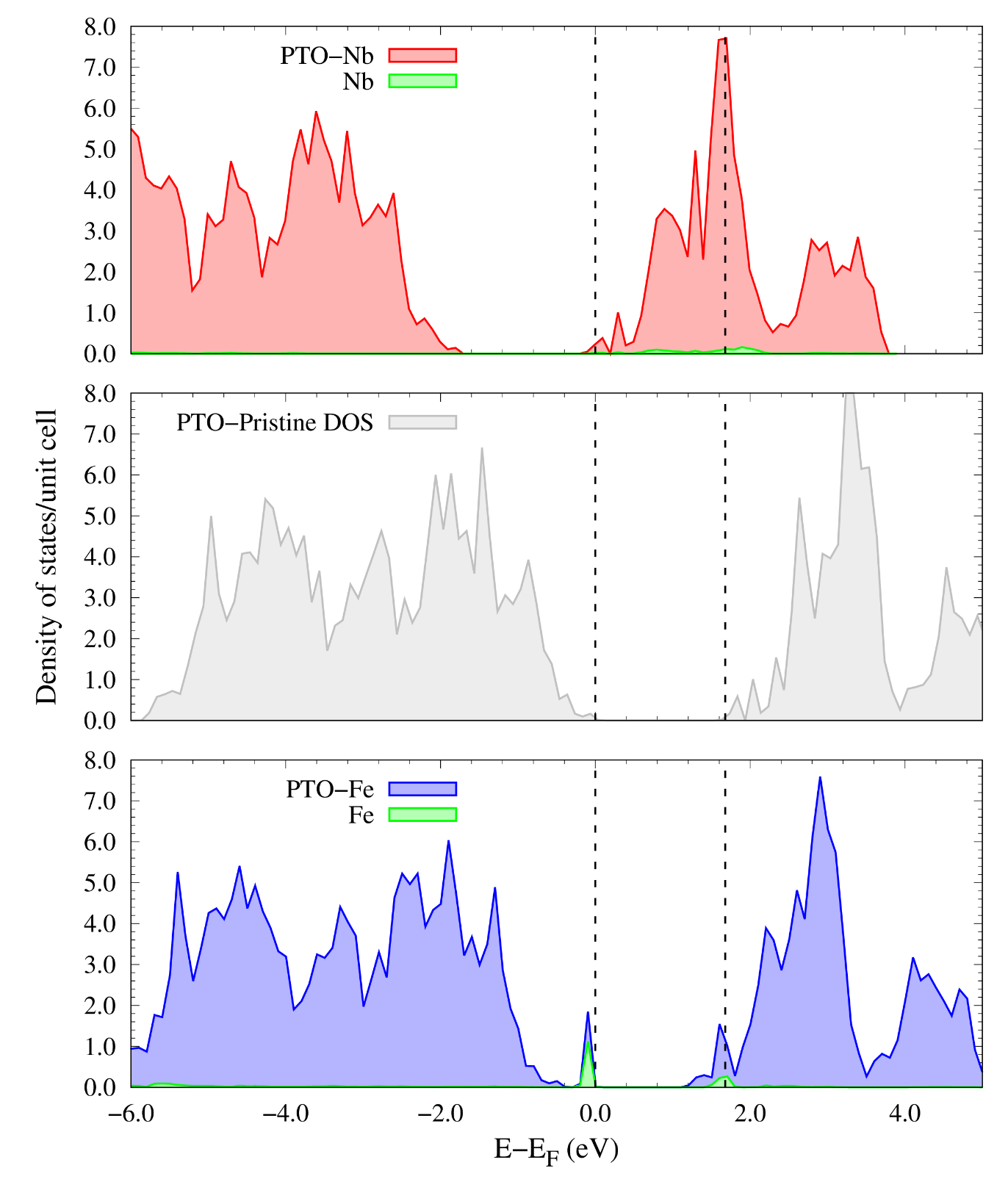


**Figure SM13.** Total density of states for PTO doped with Nb (top panel), pristine (middle panel) and with Fe doping (bottom panel). The green plots are the density of states of the doping atom alone. The dotted lines represent the conduction band minimum and the valence band maximum for the pristine PTO case.


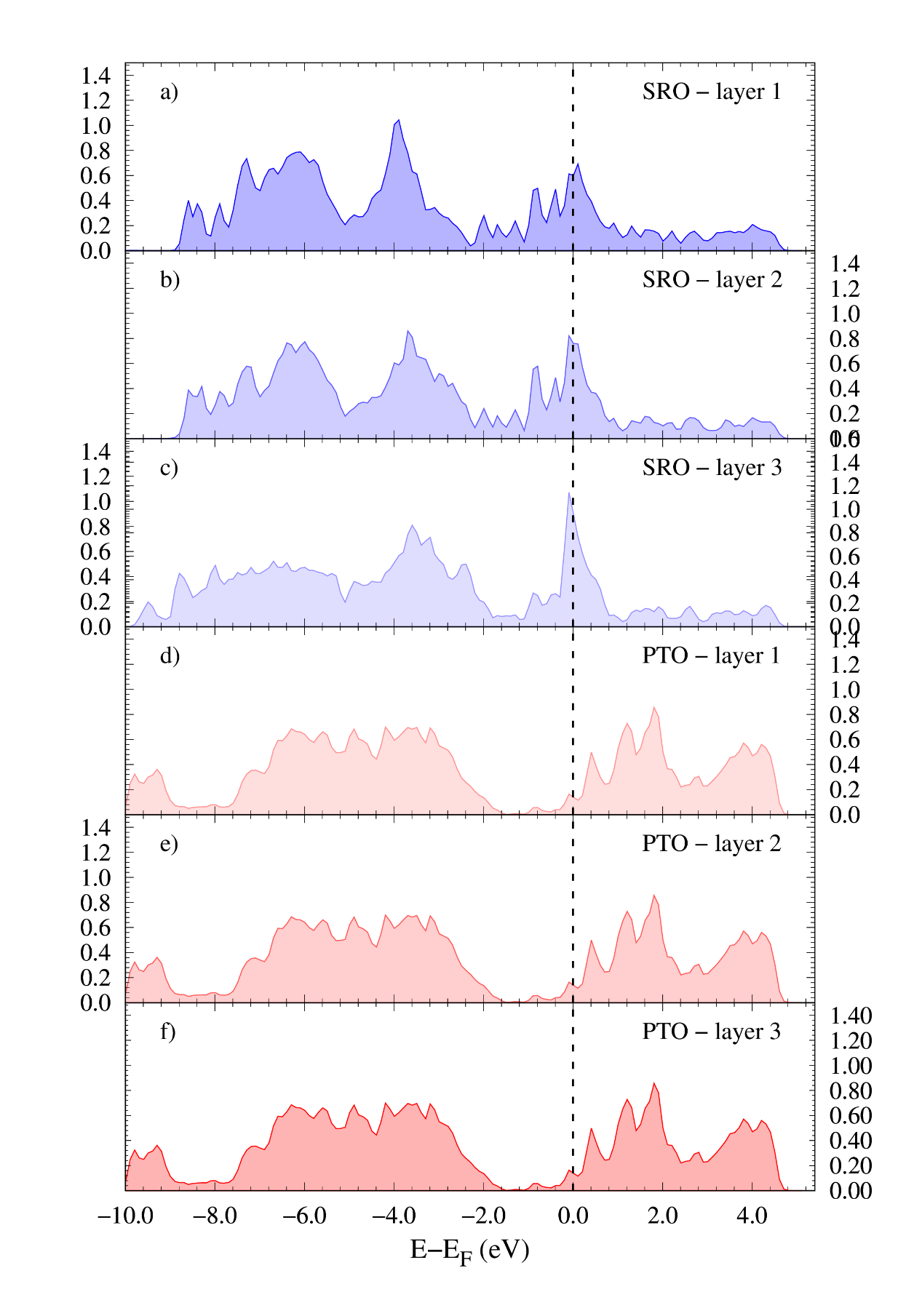


**Figure SM14** Layer by layer density of states per unit cell for the SRO/pristine-PTO structure. The interface is located between panels c) and d). The dotted line represents the Fermi level of the entire structure.


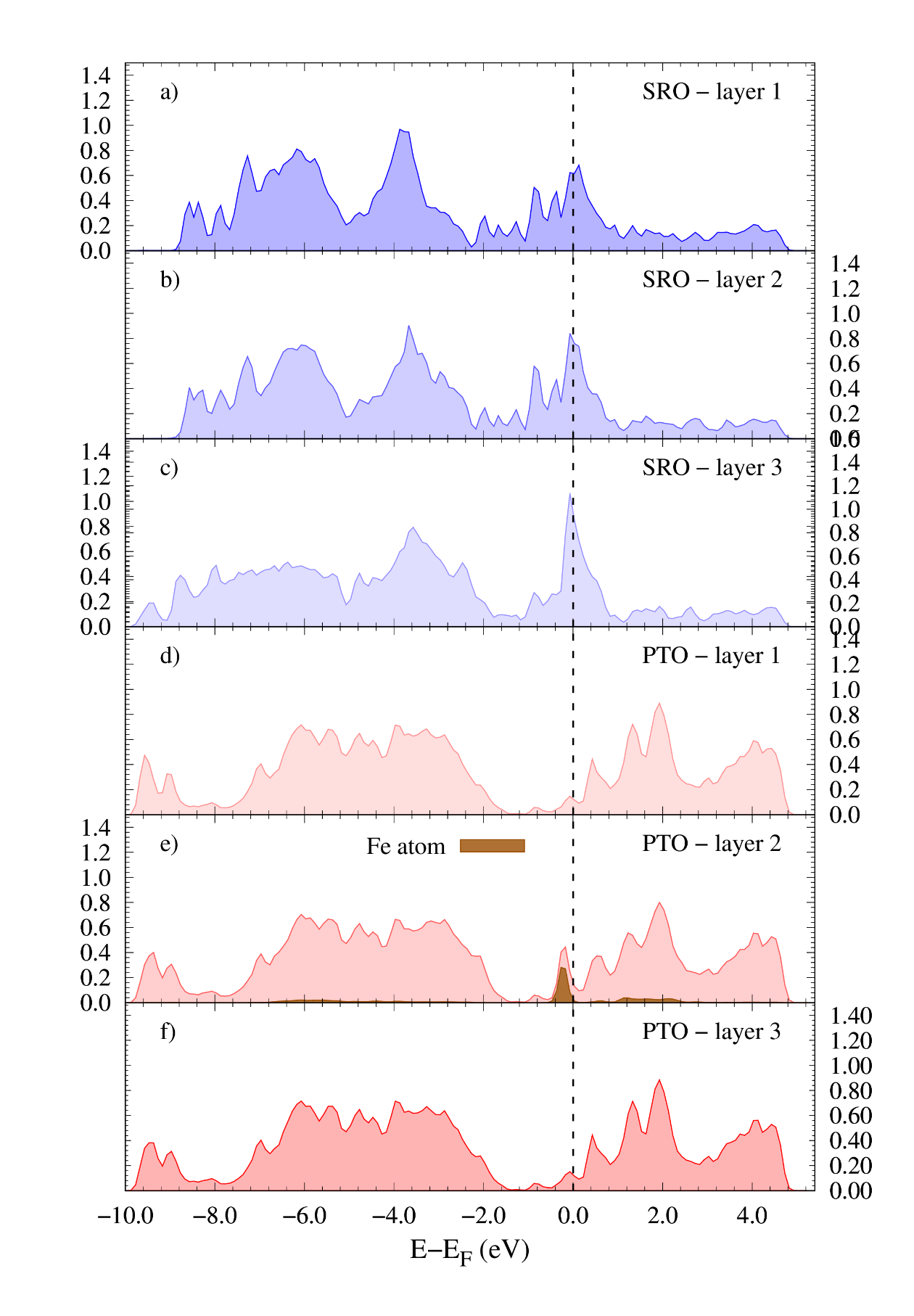


**Figure SM15** Layer by layer density of states per unit cell for the SRO/PTO-Fe structure. The interface is located between panels c) and d). The dotted line represents the Fermi level of the entire structure. The DOS for the Fe atom is shown in brown colour.


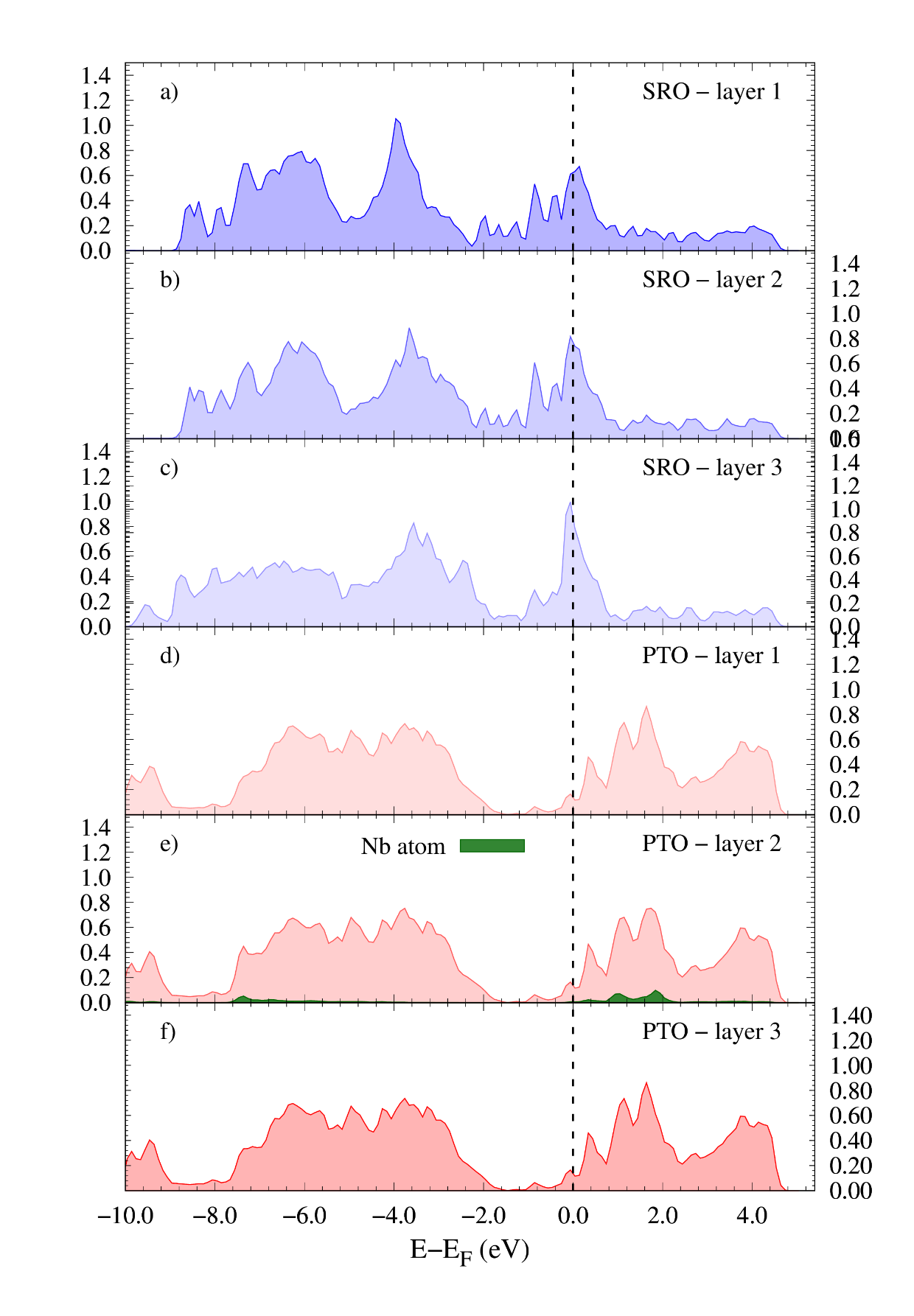


**Figure SM16** Layer by layer density of states per unit cell for the SRO/PTO-Nb structure. The interface is located between panels c) and d). The dotted line represents the Fermi level of the entire structure. The DOS for the Nb atom is shown in green colour.

**Caveats**

The super-cell approach for the study of very low doping concentrations requires considerable resources (RAM memory is the principal requirement!) in order to obtain good converged results in a reasonable amount of time. For this reason, it was chosen not to relax the internal coordinates of the SRO/PTO interfaces. An interface between two materials will introduce a perturbation in its vicinity. In order to be able to extract an eventual potential barrier the best method introduced by Van de Walle and Martin^14^ requires that the two materials on either side have a sufficient thickness such that far away from the interface the materials regain their bulk-like properties. This requirement is almost impossible to be satisfied in this case for two reasons: the SRO electrode must have a thickness of at least 5 unit cells in the direction perpendicular to the interface and also the effect of the ferroelectric polarization in the PTO require around 7 or 8 unit cells to regain the bulk behaviour^15^. All in all, this means that a very large structure is required and even if such a thing would be possible to calculate, several other methods must be introduced in order to be able to obtain a physical alignment of the energy bands at the interface.

Apart from the conclusions that can be drawn from the bulk calculations in **Fig. S13** that Fe and Nb atom doping may lead to a p-type or an n-type characteristic, respectively, the interface calculations are not reliable enough to draw meaningful conclusions for these structures.

**References**

1. Joseph, J., Vimala, T. M., Sivasubramanian, V. & Murthy, V. R. K. Structural investigations on Pb(ZrxT1−x)O_3_ solid solutions using the X-ray Rietveld method. *J. Mater. Sci.* **35**, 1571–1575 (2000).

2. https://www.webelements.com/.

3. Pintilie, L. & Alexe, M. Metal-ferroelectric-metal heterostructures with Schottky contacts. I. Influence of the ferroelectric properties. *J. Appl. Phys.* **98**, 124103–8 (2005).

4. Simmons, J. G. Effect of deep traps on the barrier heights of metal-insulator-metal tunnel junctions. *Phys. Rev. Lett.* **23**, 297 (1969).

5. Pintilie, L., Vrejoiu, I., Hesse, D., LeRhun, G. & Alexe, M. Ferroelectric polarization-leakage current relation in high quality epitaxial Pb(Zr,Ti)O_3_ films. *Phys. Rev. B* **75**, 104103 (2007).

6. Chirila, C. *et al.* Comparison between the ferroelectric/electric properties of the PbZr_0.52_Ti_0.48_O_3_ films grown on Si (100) and on STO (100) substrates. *J. Mater. Sci.* **50**, 3883–3894 (2015).

7. Giannozzi, P. *et al.* QUANTUM ESPRESSO: a modular and open-source software project for quantum simulations of materials. *J. Phys: Cond. Matter* **21**, 395502 (2009).

8. Perdew, J. P., Burke, K. & Ernzerhof, M. Generalized gradient approximation made simple. *Phys. Rev. Lett.* **77**, 3865 (1996).

9. Perdew, J. P. *et al.* Restoring the Density-Gradient Expansion for Exchange in Solids and Surfaces. *Phys. Rev. Lett.* **100**, 136406 (2008).

10. van Setten, M. J. *et al.* The PseudoDojo: Training and grading a 85 element optimized norm-conserving pseudopotential table. *Computer Phys. Commun.* **226**, 39–54 (2018).

11. Monkhorst, H. J. & Pack, J. D. Special points for Brillouin-zone integrations. *Phys. Rev. B* **13**, 5188 (1976).

12. Mestric, H. *et al.* The nature of iron-oxygen vacancy defect centers in PbTiO_3_. *Phys. Rev. B* **71**, 134109 (2005).

13. Stengel, M., Aguado-Puente, P., Spaldin, N. & Junquera, J. Band alignment at metal/ferroelectric interfaces: Insights and artifacts from first principles. *Phys. Rev. B* **83**, 235112 (2011).

14. Van de Walle, C. G. & Martin, R. M. Theoretical study of band offsets at semiconductor interfaces. *Phys. Rev. B* **35**, 8154–8165 (1987).

15. Dorin, R., Filip, L. D., Pintilie, L., Butler, K. T. & Plugaru, N. Designing functional ferroelectric interfaces from first-principles: dipoles and band bending at oxide heterojunctions. *New J. Phys.* **21**, 113005 (2019).
